# Supplementary material for: Pharmacological Bypass of Cockayne Syndrome B Function in Neuronal Differentiation
Source: Cell Rep. 2016 Mar 10;14(11):2554–61. doi: 10.1016/j.celrep.2016.02.051 (PMC4806223; doi:10.1016/j.celrep.2016.02.051)
Supplement: Document S1. Figures S1–S8 [file mmc1.pdf]

Cell Reports, Volume *14*

Supplemental Information

Pharmacological Bypass of Cockayne Syndrome B Function in Neuronal Differentiation

Yuming Wang, Jace Jones-Tabah, Probir Chakravarty, Aengus Stewart, Alysson Muotri, Rebecca R. Laposa, and Jesper Q. Svejstrup

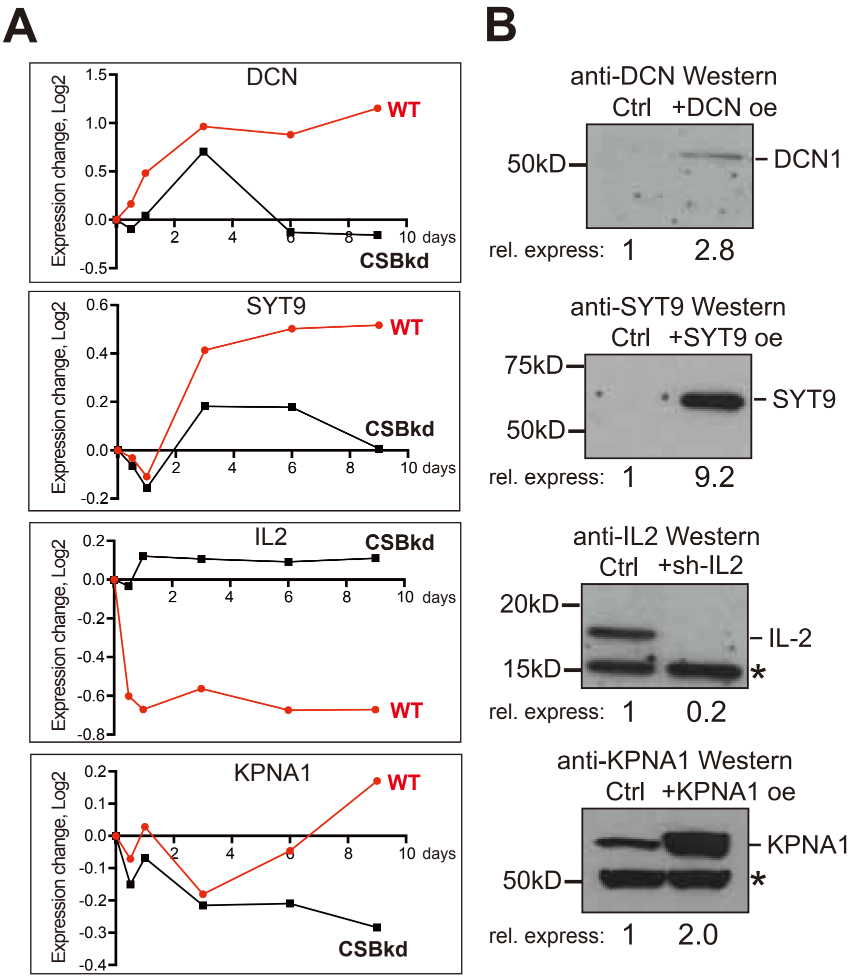
A
B
DCN
SYT9
IL2
KPNA1
WT
CSBkd
Expression change, Log2
days
anti-DCN Western
Ctrl +DCN oe
50kD
DCN1
rel. express: 1 2.8
anti-SYT9 Western
Ctrl +SYT9 oe
75kD
50kD
SYT9
rel. express: 1 9.2
anti-IL2 Western
Ctrl +sh-IL2
20kD
15kD
IL-2
rel. express: 1 0.2
anti-KPNA1 Western
Ctrl +KPNA1 oe
50kD
KPNA1
rel. express: 1 2.0

Figure S1

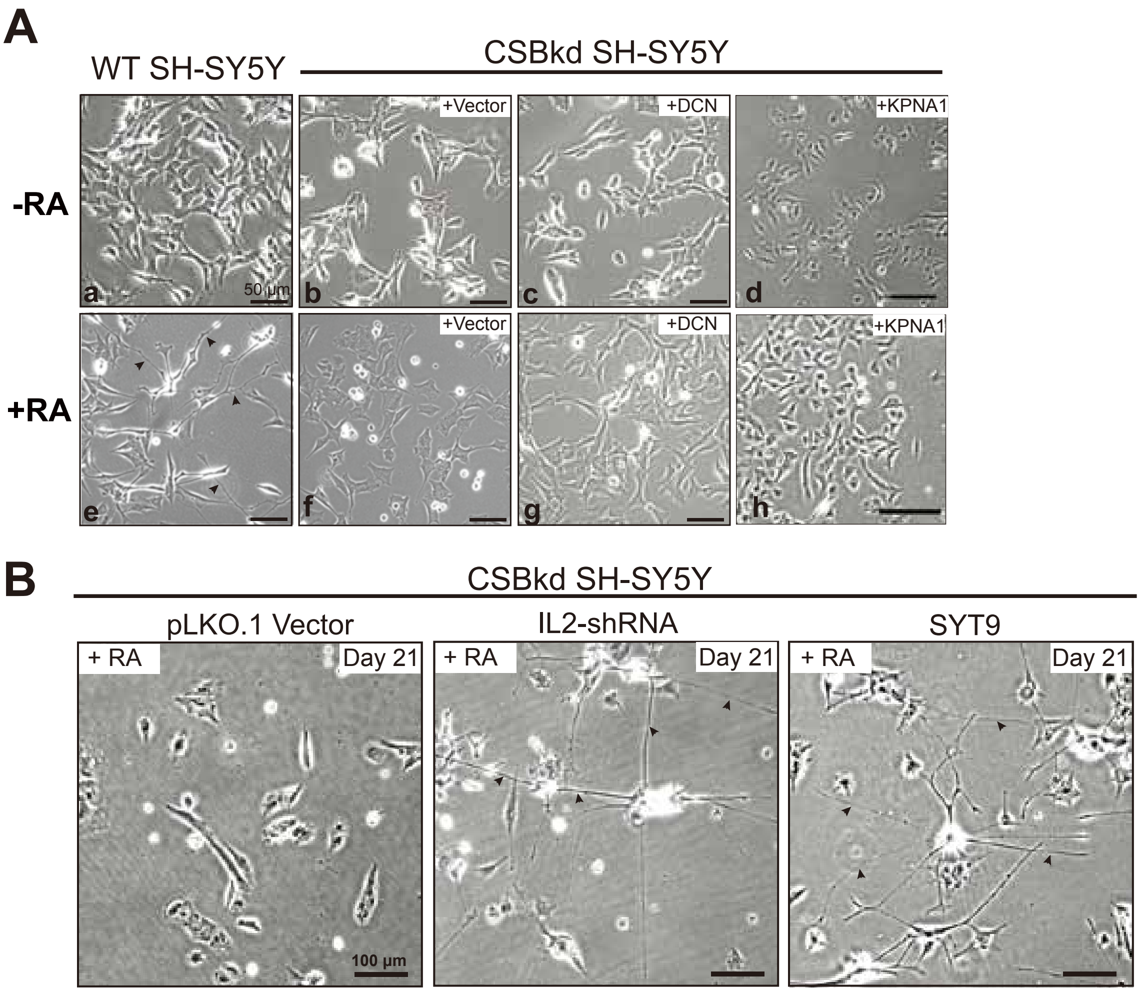
A
WT SH-SY5Y
CSBkd SH-SY5Y
-RA
+RA
+Vector
+DCN
+KPNA1
50 µm
a
b
c
d
e
f
g
h
B
CSBkd SH-SY5Y
pLKO.1 Vector
IL2-shRNA
SYT9
+ RA
Day 21
100 µm

Figure S2

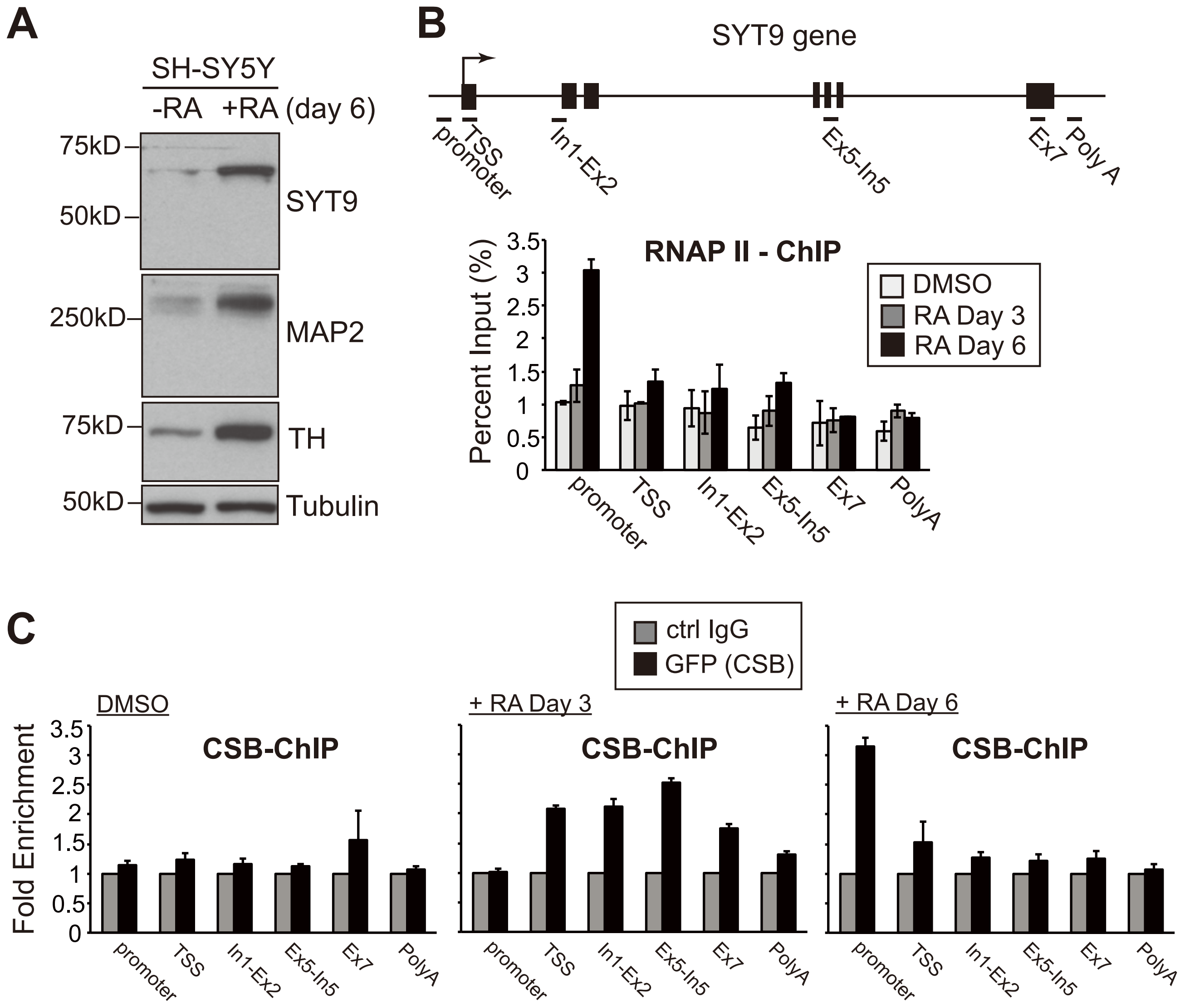
A
SH-SY5Y
-RA
+RA (day 6)
75kD
50kD
SYT9
250kD
MAP2
75kD
TH
50kD
Tubulin
B
SYT9 gene
promoter
TSS
In1-Ex2
Ex5-In5
Ex7
PolyA
RNAP II - ChIP
Percent Input (%)
DMSO
RA Day 3
RA Day 6
C
ctrl IgG
GFP (CSB)
DMSO
+ RA Day 3
+ RA Day 6
CSB-ChIP
Fold Enrichment
promoter
TSS
In1-Ex2
Ex5-In5
Ex7
PolyA

Figure S3

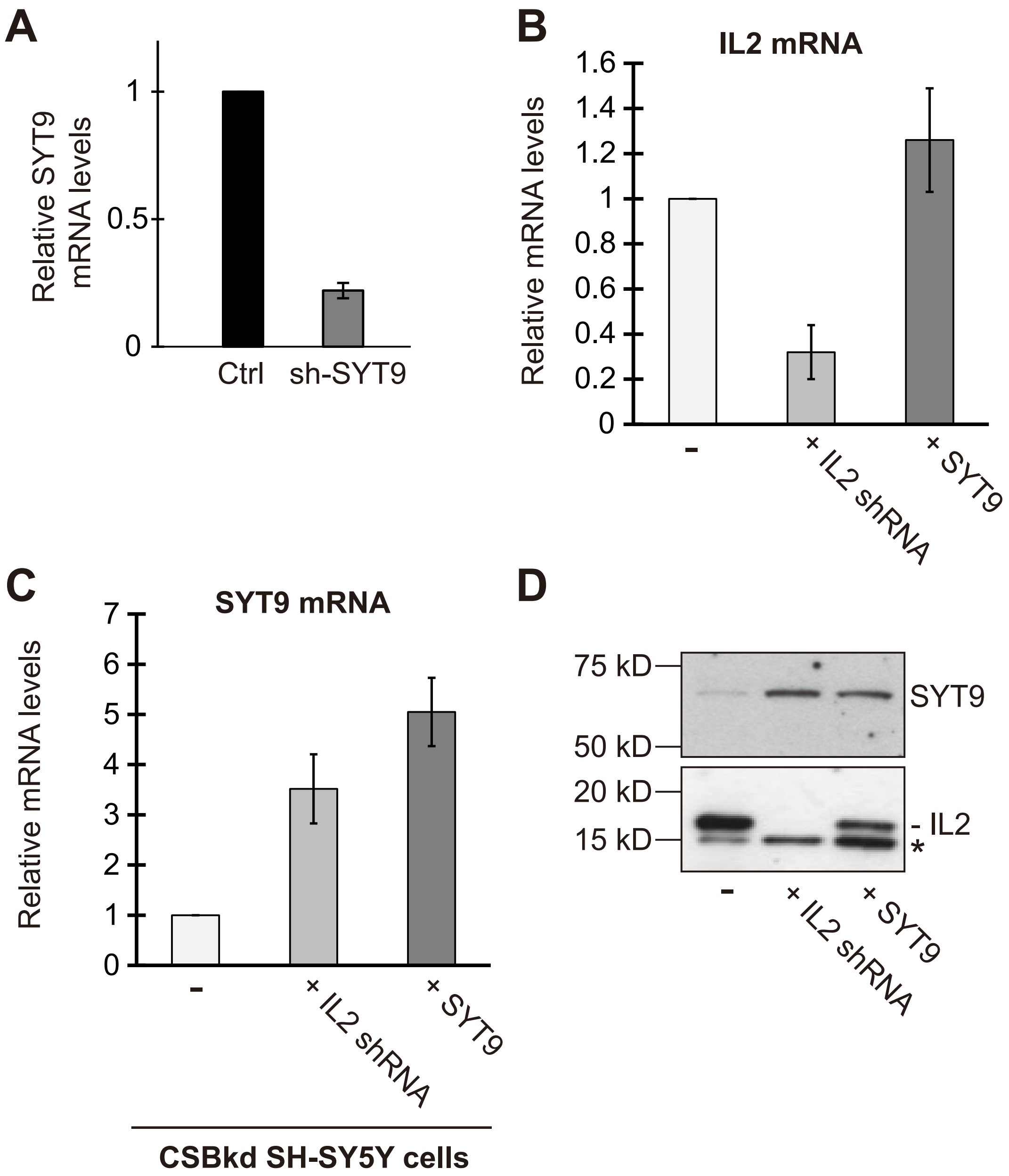

A
Relative SYT9 mRNA levels
1
0.5
0
Ctrl
sh-SYT9
B
IL2 mRNA
Relative mRNA levels
1.6
1.4
1.2
1
0.8
0.6
0.4
0.2
0
-
+ IL2 shRNA
+ SYT9
C
SYT9 mRNA
Relative mRNA levels
7
6
5
4
3
2
1
0
-
+ IL2 shRNA
+ SYT9
CSBkd SH-SY5Y cells
D
75 kD
50 kD
SYT9
20 kD
15 kD
- IL2
*
-
+ IL2 shRNA
+ SYT9

Figure S4

A

Gene symbol	Ensembl ID	Day 3 Fold-change*	Day 3 FDR-adjusted *P* value	Day 6 Fold-change*	Day 6 FDR-adjusted *P* value
SYT9	ENSG00000170743	6.16	4.32e-06	6.22	5.45e-07
ASPM	ENSG00000066279	2.61	8.30e-05	3.22	3.61e-06
EGFR	ENSG00000146648	2.14	0.000190	2.07	0.000115
SYT4	ENSG00000132872	2.28	0.001522	2.68	0.000174
GRIK4	ENSG00000149403	-	-	3.13	0.000195
PHOX2B	ENSG00000109132	-	-	2.07	0.000279
NEUROG2	ENSG00000178403	3.67	0.007165	6.17	0.000343
VPS16	ENSG00000215305	1.65	0.003025	1.82	0.000420
DBH	ENSG00000123454	2.40	0.000445	1.65	0.019542
NEUROG3	ENSG00000122859	2.37	0.010554	3.35	0.000604
LZTS1	ENSG00000061337	-	-	2.52	0.000708
ISL1	ENSG00000016082	-	-	2.08	0.000855
PTPN11	ENSG00000179295	2.12	0.001005	-	-
RND1	ENSG00000172602	-	-	2.13	0.001083
PEBP1	ENSG00000089220	1.74	0.012385	2.09	0.001213
TRKB	ENSG00000148053	-	-	2.38	0.001462
MYH10	ENSG00000133026	1.53	0.010630	1.71	0.001462
TUBB4	ENSG00000104833	2.02	0.002253	1.60	0.023046
CALCA	ENSG00000110680	2.25	0.004745	2.28	0.002937
CNP	ENSG00000173786	1.64	0.003020	-	-
ASCL1	ENSG00000139352	-	-	1.86	0.003074
SEMA4D	ENSG00000187764	1.71	0.020099	1.60	0.005496
ATF5	ENSG00000169136	-	-	2.20	0.006120
PHGDH	ENSG00000092621	-	-	2.00	0.006922
BDNF	ENSG00000176697	-	-	2.20	0.007249
TIMP2	ENSG00000035862	1.54	0.022951	1.59	0.007260
NEFH	ENSG00000100285	-	-	2.13	0.007971
EPHB3	ENSG00000182580	-	-	2.27	0.007991
CCND2	ENSG00000118971	2.28	0.011732	1.98	0.029496
NGRN	ENSG00000182768	-	-	1.87	0.012257
NRP2	ENSG00000118257	3.04	0.012886	-	-
NFASC	ENSG00000163531	-	-	1.72	0.013841
CDK5RAP2	ENSG00000136861	-	-	1.68	0.014350
FARP2	ENSG00000006607	-	-	1.80	0.016380
SERPINF1	ENSG00000132386	1.81	0.023189	2.00	0.021479
TUBB2B	ENSG00000137285	1.54	0.021551	-	-
KLF7	ENSG00000118263	1.61	0.044933	1.70	0.023644
PSEN1	ENSG00000080815	1.91	0.043057	1.97	0.031566
SEMA6A	ENSG00000092421	-	-	1.60	0.034519
EPHB2	ENSG00000133216	-	-	1.64	0.039308
MIB1	ENSG00000101752	1.60	0.029260	1.54	0.039811
LRRTM1	ENSG00000162951	-	-	1.56	0.049225
TGIF2	ENSG00000118707	-	-	1.54	0.049834

B

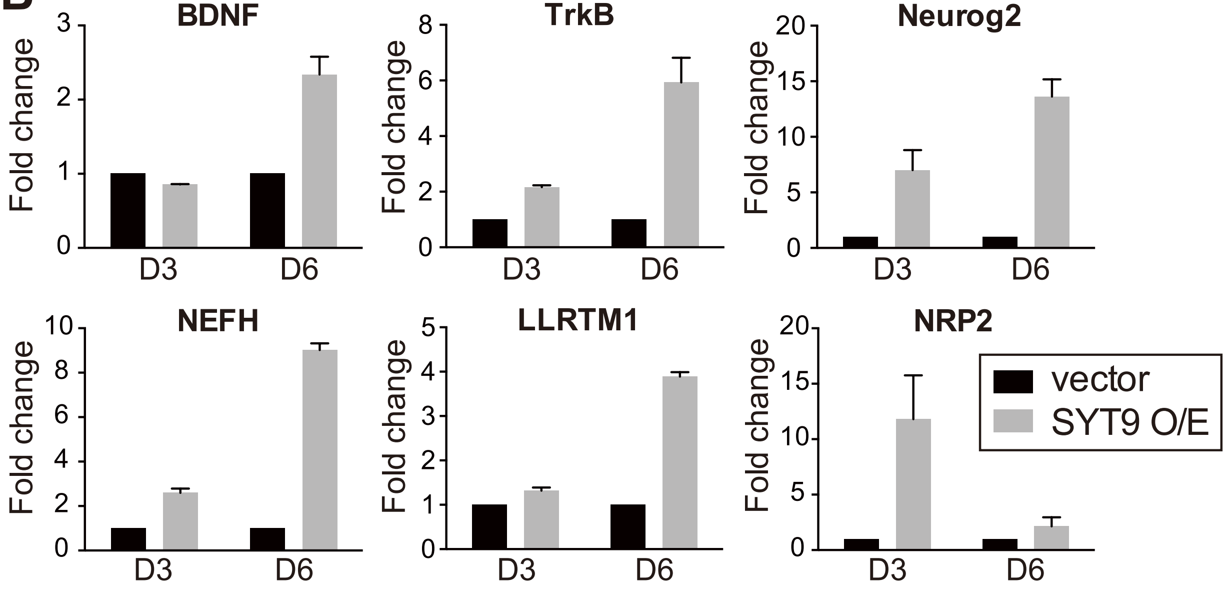

Figure S5

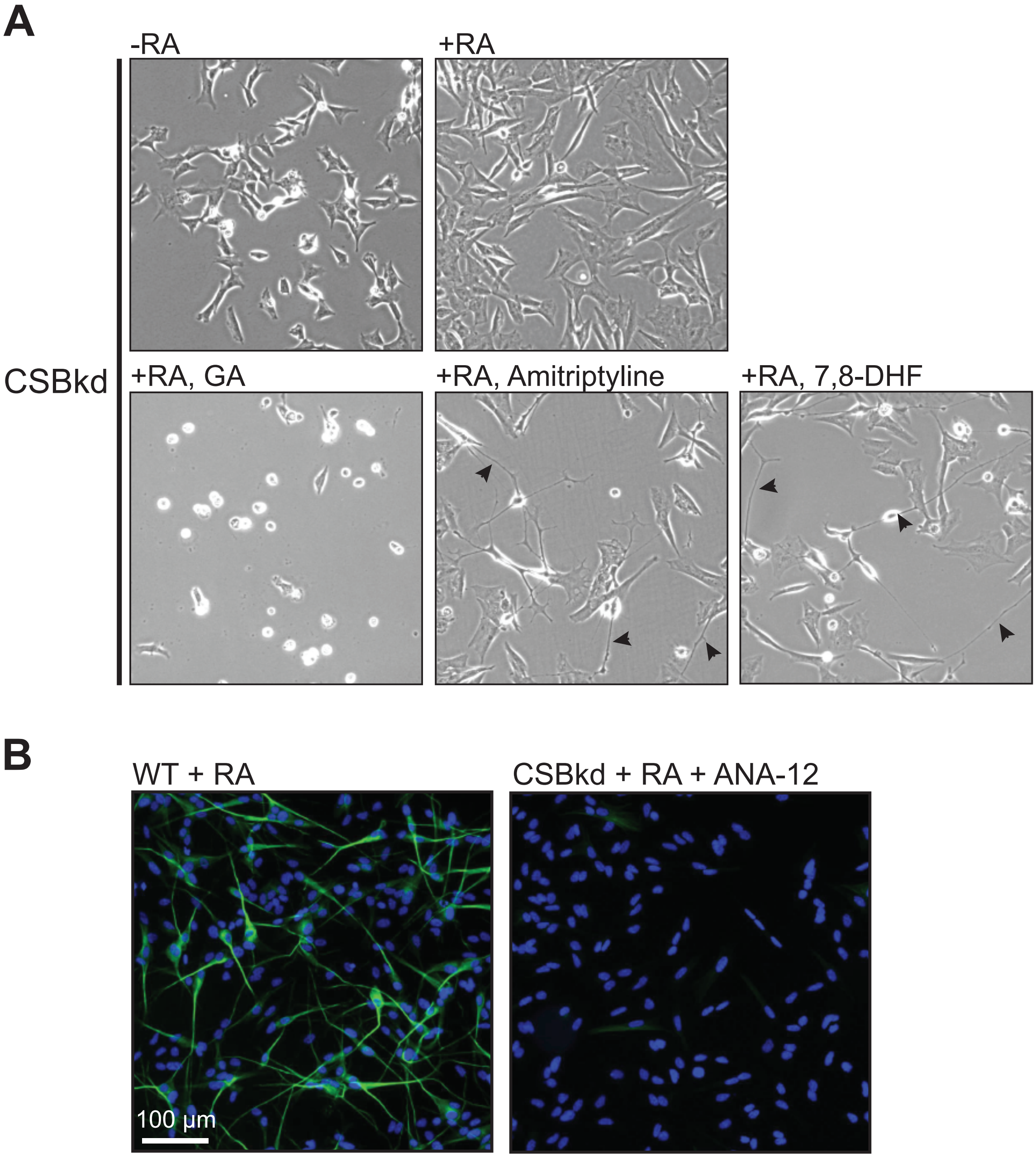
A
-RA
+RA
CSBkd
+RA, GA
+RA, Amitriptyline
+RA, 7,8-DHF
B
WT + RA
CSBkd + RA + ANA-12
100 µm

Figure S6

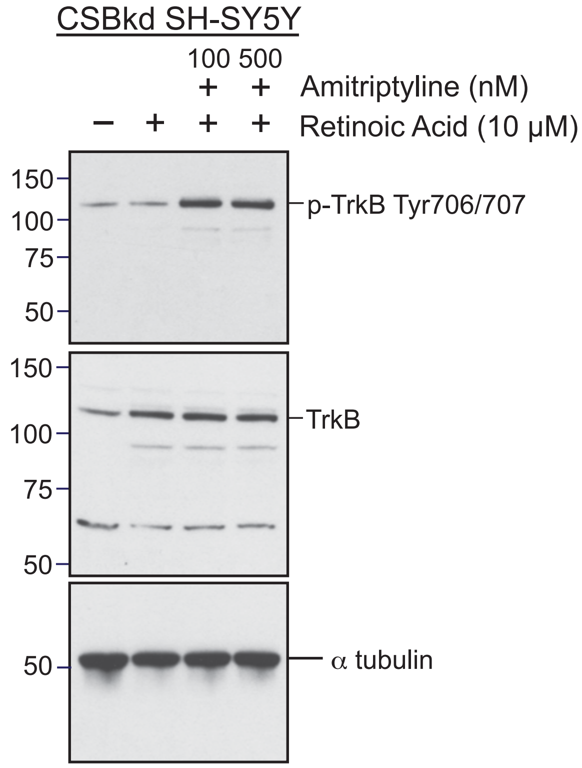

CSBkd SH-SY5Y
100 500
+ + Amitriptyline (nM)
− + + + Retinoic Acid (10 μM)
150
100
75
50
p-TrkB Tyr706/707
150
100
75
50
TrkB
50
α tubulin

Figure S7

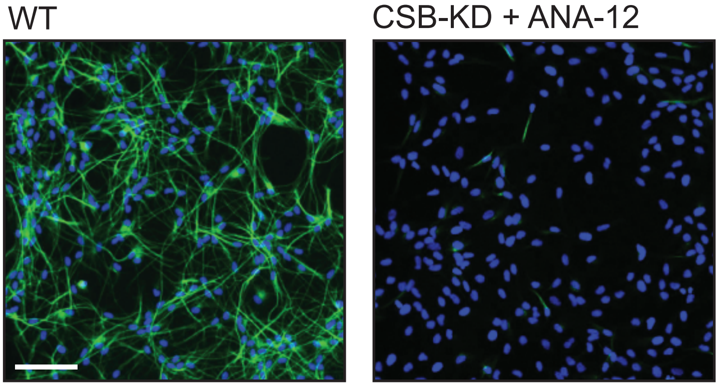

Figure S8

SUPPLEMENTARY FIGURE LEGENDS

Figure S1, related to Figure 1. qRT-PCR validation of selected genes during differentiation of WT and CSB-depleted SH-SY5Y cells. A. Quantitative RT-PCR of selected CSB-dependent genes identified by microarray expression analysis (Wang et al., 2014), at different times (in days) after retinoic acid addition. **B.** Western blot analysis of total extracts from CSB-depleted SH-SY5Y cells transduced with lentivirus carrying DCN-cDNA, SYT9-cDNA, IL2-shRNA, or KPNA1-DNA, and treated with retinoic acid for 6 days. Asterisks indicate non-specific bands.

Figure S2, related to Figure 1. Ectopic SYT9 expression or IL2 knockdown can restore neuritogenesis in CSB-depleted SH-SY5Y cells. A. CSB-depleted SH-SY5Y cells transduced with lentiviral particles containing empty vector, DCN-cDNA, or KPNA1-cDNA were treated with 10 μM RA, and phase contract images were taken on day 6 of differentiation. Left panel represents WT control cells. Arrowheads denote examples of neurite outgrowth. Scale bar = 50 μm. **B.** CSB-depleted SH-SY5Y cells expressing IL2-shRNA or SYT9-cDNA were cultured in differentiation media containing 10 μM RA. Phase contract images were taken on day 21. Arrowheads denote examples of neurite outgrowth. Scale bar = 100 μm.

Figure S3, related to Figure 1. Evidence that SYT9 induction is regulated by CSB at the level of transcription. A. Western blot analysis of whole cell lysates from undifferentiated (-RA) and differentiated (+RA) WT SH-SY5Y cells with antibodies against SYT9, MAP2, TH, and alpha tubulin. **B.** Upper, diagram showing the location of SYT9 primer sets used for ChIP-qPCR in cells expressing CSB-GFP.

Lower, ChIP analysis with antibody to RNAPII. **C.** CSB-GFP ChIP before and during RA treatment. Primers in Table S3.

Figure S4, related to Figure 1. Knockdown efficiency and effects thereof in SH-SY5Y cells. A. Quantification of SYT9 mRNA knockdown efficiency in SH-SY5Y cells stably transduced with lentivirus containing SYT9 shRNA. GFP-shRNA was used as control. **B.** Quantification of IL2 mRNA expression in CSB-depleted SH-SY5Y cells expressing IL2-shRNA, or SYT9 cDNA. Total RNA was extracted on day 3 after lentivirus transduction. **C.** As in (**B.**) but quantification of SYT9 mRNA. **D.** IL2 and SYT9 proteins levels, detected by Western blotting. Asterisk in IL2 blot indicates non-specific band.

Figure S5, related to Figure 2. SYT9-regulated genes identified by microarray analysis. A. List of genes (GO term 'Neurogenesis') that are up-regulated in CSB-depleted SH-SY5Y cells ectopically expressing SYT9 at day 3 and 6 after RA-addition. Fold-changes represent the average of two independent biological replicates. TRKB and BDNF are highlighted. **B.** RT-PCR validations in triplicate ± s.d. of each representative RNA sample. Primers used for RT-PCR in Table S3.

Figure S6, related to Figure 3. TRKB antagonists fail to rescue the differentiation defects of CSB-depleted SH-SY5Y cells. A. CSB-depleted SH-SY5Y cells were treated with either gambogic acid (GA, 100 nM), amitriptyline (100 nM) or 7,8-DHF (100 nM), together with 10 μM RA, and phase contract images were taken on day 6 of differentiation. As suggested by the pictures, GA-treated cultures suffered significant cell death. **B.** CSB-depleted SH-SY5Y cells were treated with RA

(10 μM) and ANA-12 (100 nM), and stained for Tuj1 (green) and DAPI (blue) on day 6 after treatment. WT cells (GFP-shRNA) are shown in the left panel for comparison. Scale bar = 100 μm.

Figure S7, related to Figure 3. Effects of amitriptyline on the expression of TRKB and phospho-TrkB. Western blot analysis of total extracts from CSB-depleted SH-SY5Y cells treated with RA (10 μM) and amitriptyline (100 nM or 500 nM) for 6 days before lysis. Upper panel, pTrkB-specific antibody.

Figure S8, related to Figure 4. ANA-12 failed to rescue the defects of CSB-KD ReNcell VM cell differentiation. WT and CSB-depleted ReNcell VM cells treated with ANA-12 (100 nM) were stained for Tuj1 (green) and DAPI (blue) on week 2 after serum withdrawal. WT cells treated with RA are shown for comparison. Scale bar = 100 μm.

Supplementary Tables

• 3 Supplementary Tables (Table S1-S3)
Table S1 is related to Figure 2: **Up- and down-regulated genes in CSBkd SH-SY5Y cells ectopically expressing SYT9**

Table S2 is related to Figure 3: **Genes that are significanctly up- or down-regulated in CS patients' cerebrum**

Table S3 is related to Experimental procedures: **Primers used for qRT-PCR validation of microarray data**
