# Supplementary material for: Pharmacological Bypass of Cockayne Syndrome B Function in Neuronal Differentiation
Source: Cell Rep. 2016 Mar 10;14(11):2554–61. doi: 10.1016/j.celrep.2016.02.051 (PMC4806223; doi:10.1016/j.celrep.2016.02.051)
Supplement: Document S2. Article plus Supplemental Information [file mmc5.pdf]

Cell Reports

Report

Pharmacological Bypass of Cockayne Syndrome B Function in Neuronal Differentiation

Graphical Abstract

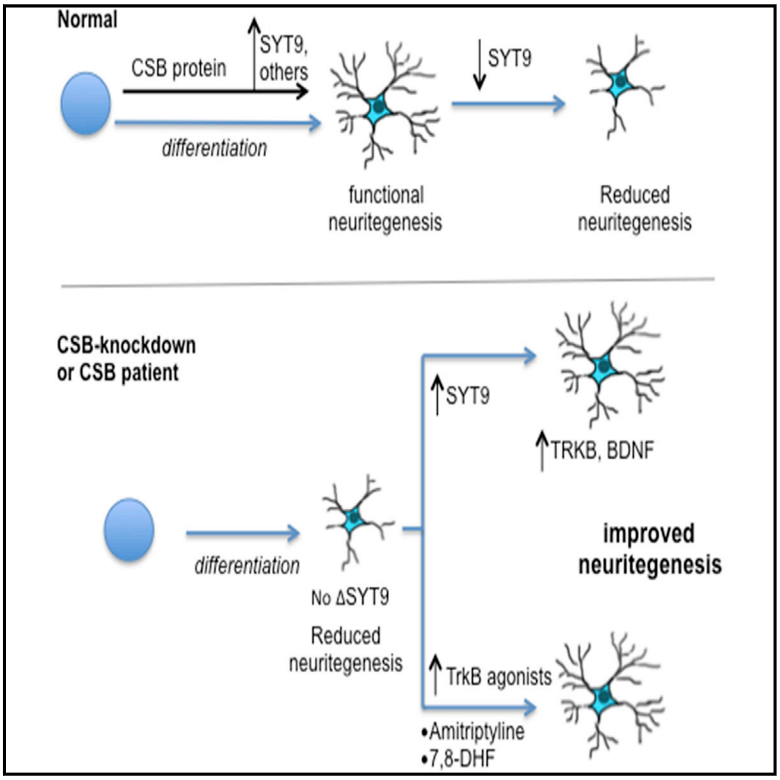

Highlights

- Neuritogenesis defects in CS cell lines can be overcome by overexpression of SYT9
- SYT9 is crucial for neuritogenesis and involved in neurotrophin (BDNF) signaling
- Neuritogenesis defects in CS cell lines can be overcome by BDNF treatment
- They can also be overcome by treatment with amitriptyline, an FDA-approved BDNF mimic

Authors

Yuming Wang, Jace Jones-Tabah, Probir Chakravarty, Aengus Stewart, Alysson Muotri, Rebecca R. Laposa, Jesper Q. Svejstrup

Correspondence

jesper.svejstrup@crick.ac.uk

In Brief

Wang et al. show that Cockayne syndrome cell lines have defects in gene regulatory loops, resulting in sub-optimal neurotrophin signaling and explaining their defects in neurogenesis. These defects can be overcome by Synaptotagmin 9 overexpression or by treatment with NTRK2 (TrkB) agonists, pointing to future disease intervention.

Accession Numbers

GSE58072

Wang et al., 2016, Cell Reports *14*, 2554–2561
March 22, 2016 ©2016 The Authors
http://dx.doi.org/10.1016/j.celrep.2016.02.051

OPEN ACCESS
CellPress

Cell Reports
Report

Pharmacological Bypass of Cockayne Syndrome B Function in Neuronal Differentiation

Yuming Wang,[1,6] Jace Jones-Tabah,[2] Probir Chakravarty,[3] Aengus Stewart,[3] Alysson Muotri,[4,5] Rebecca R. Laposa,[2] and Jesper Q. Svejstrup[1,*]
[1]Mechanisms of Transcription Laboratory, Clare Hall Laboratories, The Francis Crick Institute, South Mimms, Hertfordshire EN6 3LD, UK
[2]Department of Pharmacology and Toxicology, University of Toronto, 1 King's College Circle, Toronto, ON M5S 1A8, Canada
[3]Bioinformatics & Biostatistics Group, The Francis Crick Institute, 44 Lincoln's Inn Fields, London WC2A 3LY, UK
[4]Department of Pediatrics
[5]Department of Cellular and Molecular Medicine
University of California, San Diego, 2800 Torrey Pines Scenic Drive, La Jolla, CA 92037, USA
[6]Present address: State Key Laboratory of Ophthalmology, Zhongshan Ophthalmic Center, Sun Yat-sen University, Guangzhou 510060, China
*Correspondence: jesper.svejstrup@crick.ac.uk
http://dx.doi.org/10.1016/j.celrep.2016.02.051
This is an open access article under the CC BY license (http://creativecommons.org/licenses/by/4.0/).

SUMMARY

Cockayne syndrome (CS) is a severe neurodevelopmental disorder characterized by growth abnormalities, premature aging, and photosensitivity. Mutation of Cockayne syndrome B (CSB) affects neuronal gene expression and differentiation, so we attempted to bypass its function by expressing downstream target genes. Intriguingly, ectopic expression of Synaptotagmin 9 (SYT9), a key component of the machinery controlling neurotrophin release, bypasses the need for CSB in neuritogenesis. Importantly, brain-derived neurotrophic factor (BDNF), a neurotrophin implicated in neuronal differentiation and synaptic modulation, and pharmacological mimics such as 7,8-dihydroxyflavone and amitriptyline can compensate for CSB deficiency in cell models of neuronal differentiation as well. SYT9 and BDNF are downregulated in CS patient brain tissue, further indicating that sub-optimal neurotrophin signaling underlies neurological defects in CS. In addition to shedding light on cellular mechanisms underlying CS and pointing to future avenues for pharmacological intervention, these data suggest an important role for SYT9 in neuronal differentiation.

INTRODUCTION

Cockayne syndrome (CS) is a hereditary, multisystem disease, characterized by neurological and developmental impairment as well as sun sensitivity and progeroid-like features (Brooks, 2013). Despite the accumulating knowledge on the role of the main causative gene, *Cockayne syndrome B* (*CSB*), in the control of various biological processes, an understanding of its role in CS has been missing. Indeed, a lack of effective mechanism-based therapeutic approaches means that CS is devastating, with a large number of symptoms related to nervous system deficiencies and often resulting in death within the first decade of life.

CSB is a member of the SWI2/SNF2 family of ATP-dependent chromatin remodeling factors (Troelstra et al., 1992; Citterio et al., 1998), and its mutation accounts for the vast majority (~80%) of CS cases. Certain mutations in the CS genes can also give rise to the clinically less severe UV-sensitive syndrome (UVSS) (reviewed in Spivak, 2005). CSB is a multi-functional factor. Indeed, it has been implicated not only in transcription-coupled nucleotide excision repair (TC-NER) and base excision repair but also in mitochondrial function and regulation of transcription (reviewed in Weidenheim et al., 2009; Brooks, 2013; Scheibye-Knudsen et al., 2013; Vélez-Cruz and Egly, 2013; Vermeulen and Fousteri, 2013).

Studies of the molecular mechanisms underlying human disease have often relied on animal models. Intriguingly, while mice lacking *Csb* show sun sensitivity, they do not display the same severe growth retardation, neurologic defects, or high early mortality that is characteristic of human CS patients (van der Horst et al., 1997). Thus, the mouse model appears to provide an excellent model for UVSS, while it is arguably somewhat less helpful for our understanding of CS. Interestingly, recent studies in different human cell differentiation systems showed that a lack of functional CSB represents a barrier to neuronal cell differentiation (Ciaffardini et al., 2014; Wang et al., 2014). For example, we reported that direct reprogramming of CS fibroblasts to neurons is defective and that little or no differentiation of neuroblastoma cells to neuron-like cells was observed in the absence of CSB (Wang et al., 2014). This correlated with gene expression defects in neuronal gene networks (which were not observed in the mouse), suggesting that transcription defects, rather than DNA repair- or mitochondrial defects, underlie the severe neurologic symptoms of CS (Wang et al., 2014). However, the mechanism and important downstream implementing factors remained unknown. Crucially, without knowledge of these

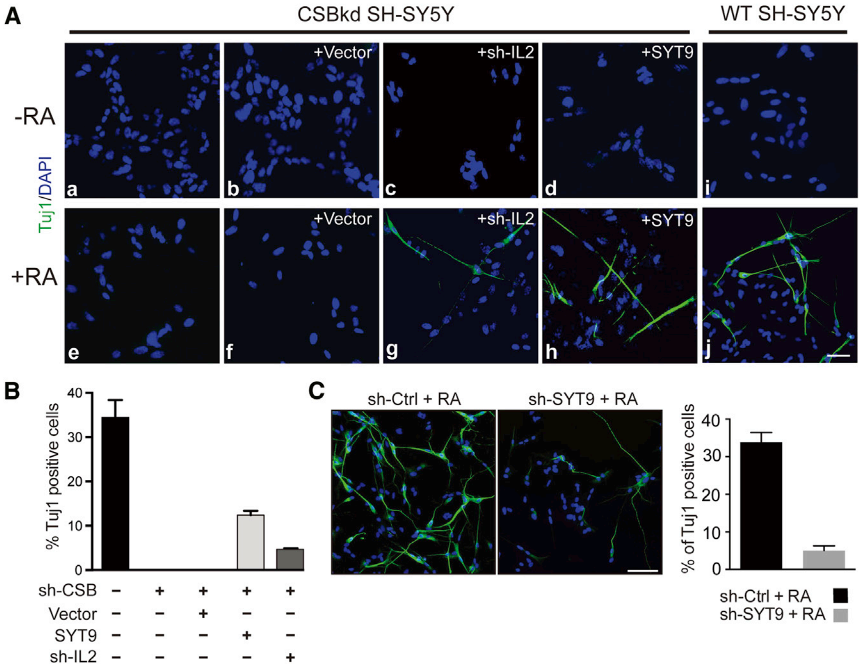

Figure 1. Ectopic SYT9 Expression Can Bypass the Requirement for CSB Function during Differentiation of SH-SY5Y Cells

(A) CSB-depleted SH-SY5Y cells were transduced with lentivirus carrying control, IL2 shRNA, or SYT9 cDNA. On day 2 after transduction, cells were induced to differentiate by RA addition. Tuj1 (green) and DAPI (blue) staining was performed on day 6 after RA treatment. j depicts differentiation in WT cells for comparison (compare to e and f). Scale bar, 100 μm.

(B) Differentiation quantitated by Tuj1-positive cells. Bar graphs represent means ± SD (n > 500 cells per data point).

(C) Differentiation of WT (sh-Ctrl) and SYT9-depleted (sh-SYT9) cells, as in (A) and (B). Scale bar, 100 μm. Bar graphs represent means ± SD (n > 500 cells per data point).

factors, mechanism-based therapeutic approaches remain elusive.

Here, we used different human cell models to comprehensively examine the connection between CSB-dependent gene expression and neurogenesis. This led to the discovery that brain-derived neurotrophic factor (BDNF), or its pharmacologic mimics, can partially bypass the requirement for CSB in neuronal differentiation. These findings provide an understanding of the basic mechanisms underlying CS and give hope for future disease intervention.

RESULTS

We previously showed that reprogramming of CS fibroblasts to neuron-like cells is defective and that CSB-depleted SH-SY5Y neuroblastoma cells fail to differentiate. This correlates with defects in transcriptional regulation of relevant neuronal genes. By examining the transcriptome during differentiation of SH-SY5Y cells, we identified ~100 CSB-dependent genes that display significant temporal and quantitative changes (Wang et al., 2014).

We surmised that, if gene expression deficiencies are, indeed, the cause of differentiation defects in CSB-depleted SH-SY5Y cells, rather than just correlating with them, then it might be possible to bypass CSB function by artificially altering the expression of these downstream, CSB-regulated genes. This approach would also address the relative importance of CSB in the regulation of gene expression and DNA repair for neuronal development and survival. In this endeavor, we restricted our focus to genes whose expression changed in a consistent manner during neuronal differentiation, temporally and quantitatively (i.e., sustained increases or decreases), as such characteristics could be mimicked by ectopic gene expression or RNAi knockdown. From a number of candidates (Wang et al., 2014), we chose to further analyze DCN (encoding decorin, a small proteoglycan) and SYT9 (Synaptotagmin-9), which were upregulated, and IL2 (interleukin-2), which was downregulated, in wild-type (WT) but not CSB-depleted cells. As a "control," we also examined KPNA1 (karyopherin alpha-1), which varied in expression during the time course, showing modest decreases in expression at early time points, and then increased moderately 9 days after retinoic acid (RA) addition in WT cells, but not to the same extent in CSB-deficient cells (Wang et al., 2014) (Figures S1A and S1B).

Ectopic expression of DCN and KPNA1 failed to affect the differentiation defects caused by CSB depletion (Figure S2A). Remarkably, however, expressing SYT9 or knocking down IL2 partially rescued the defects in RA-dependent neurite outgrowth, as indicated by an increase in cells positive for Tuj1, a marker of neuron differentiation (Figure 1A). Indeed, SYT9 re-expression brought differentiation efficiency back to almost half that observed in WT SH-SY5Y cells (Figure 1B). We previously reported that CSB is also required for the maintenance of neurites in already differentiated SH-SY5Y cells (Wang et al., 2014). Interestingly, neurites were stable over a long time period in cells ectopically expressing SYT9 (or in which IL2 was knocked down) (Figure S2B), suggesting that the positive effect was maintained. We also confirmed that SYT9 protein levels are increased during differentiation of WT SH-SY5Y cells (Figure S3A). Moreover, RNA polymerase II (RNAPII) and CSB chromatin immunoprecipitation (ChIP) suggested that the SYT9 gene might be directly regulated by CSB during the early stages of differentiation, with CSB occupancy levels at the gene mirroring gene expression levels (Figures S3B and S3C).

SYT9 is a member of the large synaptotagmin family of vesicle proteins that control calcium-dependent exocytosis. The specific function of SYT9 has been the subject of some debate (Xu et al., 2007; Zhu et al., 2007; Dean et al., 2012), but recent results indicate that it is associated with dense core vesicle dynamics and the release of neurotrophic factors and neuropeptides (Dean et al., 2012). Consistent with an important role for SYT9

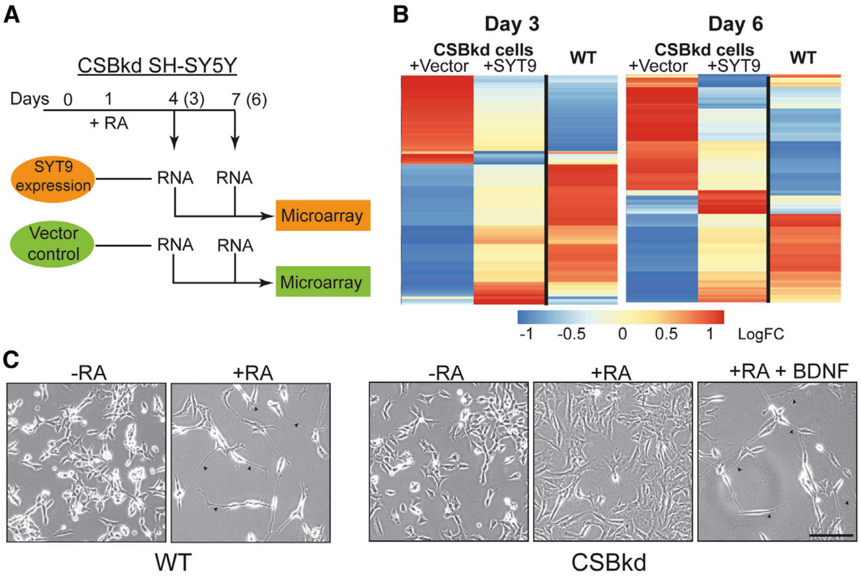

Figure 2. Ectopic SYT9 Expression "Corrects" Gene Expression Deficiencies in CSB-Depleted Cells

(A) Strategy used to identify SYT9-regulated genes during differentiation in CSB-depleted cells.

(B) Normalized relative expression, shown as heatmaps, of differentially expressed genes in CSB-depleted SH-SY5Y cells ectopically expressing SYT9. Genes with a fold change >1.5 and an adjusted p value < 0.05 relative to control (Vector) were included, with gene expression in WT cells, which were previously compared with the CSB-depleted cells (Wang et al., 2014), shown on the right at the same time points as reference. Cream color ("0") for any individual gene is the average across the three relevant samples, and other values are relative to that.

(C) WT and CSB-depleted SH-SY5Y cells were treated with RA and BDNF (3.5 nM) as indicated, and images were taken on day 6 after treatment. Arrowheads denote examples of neurite outgrowth. Scale bar, 100 μm.

in neuronal differentiation of SH-SY5Y cells, we found that short hairpin RNA (shRNA)-mediated knockdown of SYT9 significantly reduced RA-induced differentiation in these cells (Figure 1C; Figure S4A).

The ability to partially bypass CSB's function by modulating expression of SYT9 was intriguing. However, given SYT9's role as a regulator of secretory granule release, it seemed reasonable to expect that it was part of the complex signaling pathways activating the gene expression circuitries that underlie neuritogenesis. In this model, ectopic expression of SYT9 compensates for CSB loss by helping to bring about the gene expression changes required for differentiation through other means. Supporting the notion of gene regulatory networks involving SYT9, we note that IL2 may compensate for CSB inactivation, at least partially, via SYT9. Indeed, while SYT9 overexpression did not cause a reduction in IL2 expression (Figure S4B), knockdown of IL2 led to upregulation of SYT9 (Figures S4C and S4D).

To directly investigate whether gene regulatory networks are indeed (re-)activated upon SYT9 expression, we compared the transcriptome of CSB-depleted SH-SY5Y cells expressing SYT9 with that of cells carrying only the empty vector, on days 3 and 6 after RA addition (Figure 2A). Genes that were differentially expressed between these two cell lines were identified (all affected genes are listed in Table S1), and transcript levels in this group of genes were depicted next to the previously established WT expression levels at the same time points (Figure 2B). Strikingly, while the gene expression profiles changed markedly over time after RA addition, those of CSB-deficient cells that overexpressed SYT9 were consistently more similar to that of WT cells than to those of the parental CSB-depleted cells; genes that were lowly expressed in CSB-depleted cells, compared to WT, typically became more highly expressed upon SYT9 overexpression (and vice versa for the relatively highly transcribed genes in CSB-depleted cells), indicating that SYT9 overexpression partially "corrected" the expression of genes affected by CSB inactivation. This suggests that CSB and SYT9 both provide important inputs to the same gene regulatory networks, enabling an elevated SYT9 input to partially compensate for the absence of CSB.

Interestingly, when analyzing the genes whose expression was most affected by SYT9 expression, we noticed that BDNF and its receptor, tropomyosin receptor kinase B (TRKB), were both significantly upregulated upon SYT9 expression (Figures S5A and S5B). As a master regulator of neuronal survival, differentiation, and synaptic plasticity (Park and Poo, 2013), and a likely cargo of SYT9-regulated dense core vesicles (Dean et al., 2012), BDNF appeared an excellent candidate for further studying differentiation and neuritogenesis in CSB-depleted cells. In apparent support of this idea, addition of recombinant human BDNF bypassed the need for CSB, inducing neuritogenesis in CSB-depleted SH-SY5Y cells (Figures 2C and 3A). A similar lack of requirement for CSB during neuronal differentiation when using conditions that involve the addition of both RA and BDNF (Encinas et al., 2000) has recently been observed by others (Iyama et al., 2015).

It would be of potential clinical relevance if it were possible to pharmacologically compensate for the lack of CSB function in neuronal differentiation and maintenance. Unfortunately, the therapeutic use of BDNF is limited, due to its poor pharmacokinetic profile (Jang et al., 2010). However, small-molecule agonists of TRKB, such as 7,8-dihydroxyflavone (7,8-DHF) and amitriptyline, can mimic BDNF stimulation, and recent results suggest that these might be promising therapeutic tools for the treatment of neurological disease (Jang et al., 2010; Jiang et al., 2013; Cong et al., 2015; Zhao et al., 2015). To investigate the possible effect of BDNF mimics in bypassing CSB function, we treated CSB-depleted SH-SY5Y cells with these agonists. As controls for specificity, we also used a selective TRKA agonist, gambogic acid (GA), as well as the TRKB antagonist, ANA-12. Remarkably, the TRKB agonist-treated cells grew neurites, and the differentiation efficiency was comparable to that of CSB-deficient cells overexpressing SYT9 (Figure 3; Figure S6A). In contrast, and as expected, GA and ANA-12 failed to rescue

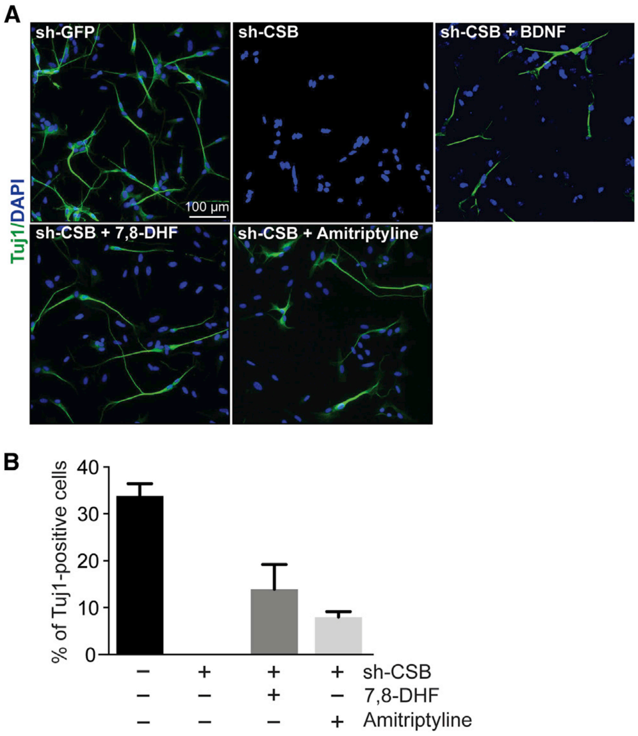

Figure 3. TrkB Agonists Can Rescue the Differentiation Defects of CSB Depletion in SH-SY5Y Cells

(A) CSB-depleted SH-SY5Y cells (CSB shRNA [shCSB]) were treated with BDNF (3.6 nM), amitriptyline (100 nM), or 7,8-DHF (100 nM) and immunostained with Tuj1 antibodies on day 6 after 10 μM RA treatment. Control-depleted SH-SY5Y cells (shGFP) are shown for comparison. Scale bar, 100 μm.

(B) Quantitation of cells expressing Tuj1. Bars represent means ± SD (n > 500 cells per data point).

the defects, with GA causing significant cell death (Figure S6B). Amitriptyline restored TRKB phosphorylation without affecting expression levels (Figure S7).

Together, the aforementioned data are consistent with the idea that CSB inactivation results in the sub-optimal expression of gene regulatory networks that are activated by BDNF during differentiation of neuroblastoma cells and that CSB function can be, at least partially, bypassed by artificially re-activating these networks, through SYT9, BDNF, or BDNF-mimicking drugs such as 7,8-DHF and amitriptyline.

Up to this point, the data were generated using neuroblastoma SH-SY5Y cells, and it could be argued that these are not an ideal or general model for neuronal differentiation and maintenance in humans. We note, however, that we previously found SYT9 and BDNF to be among the most downregulated genes in human cerebella from CS patients (Wang et al., 2014). To expand on this finding, we now compared the gene expression profiles of cerebrum samples from post-mortem CS patients and matched controls. Gratifyingly, SYT9 and BDNF were among the most significantly downregulated genes in CS patients also in this tissue (Table S2), arguing for a general effect of CSB mutation on the gene regulatory networks encompassing these factors in human brains.

To further expand on the findings in the SH-SY5Y neuroblastoma cells, we also used the human neural progenitor stem cell line, ReNcell VM, which can readily differentiate into the principal neural cell types, including neurons. Upon infection with CSB shRNA-containing lentivirus, CSB protein was barely detectable in these cells (Figure 4A). Tellingly, expression of SYT9 was dramatically increased during neuronal differentiation in WT, but not in the CSB-depleted cells (Figure 4B, left panel), suggesting that, as in SH-SY5Y cells, normal SYT9 gene activation requires CSB during neuronal differentiation of ReNcell VM. CSB was also required for the induction of neuronal differentiation marker, MAP2 (Figure 4B, right panel). More importantly, staining for neurons with Tuj1 antibody was greatly reduced in CSB-depleted ReNcell VM cells, suggesting that CSB is required for neuronal differentiation also in this cell line (Figure 4C; compare panels b and e with panels a and d). However, as previously observed in SH-SY5Y, treatment with amitriptyline partially restored differentiation (Figure 4C, c and f). In contrast, and as expected, the antagonist ANA-12 was unable to rescue the defects (Figure S8).

Fibroblast-derived, induced pluripotent stem cells (iPSCs) from patients represent an alternative approach to the study of neurodevelopment and neurodegenerative disease (Dimos et al., 2008). Recent data show that, consistent with our previous results (Wang et al., 2014), iPSCs derived from CS patient cells are partially defective in neuronal differentiation (Vessoni et al., 2016). Therefore, to further expand on the finding that CSB function can be bypassed with TrkB agonists in neuroblastoma cells and ReNcell VM cells, we derived neurons from CSB patient-derived iPSCs and cultured these for 1 week in the absence or presence of TRKB agonists amitriptyline or 7,8-DHF. Gratifyingly, an increase in neurite number and arborization in patient-derived cells by treatment with these small molecules was observed (Figures 4D–4G), consistent with the notion that increased TrkB signaling can improve neuronal differentiation in CS models.

Together, these results indicate that the effect of CSB depletion on gene regulatory networks and neuronal cells is not restricted to a particular cell type but that it has general validity, with similar results obtained in three different human cell models.

DISCUSSION

CS is a devastating disease with high mortality at a very young age and a large number of clinical symptoms related to deficiencies in neuronal development and maintenance (Brooks, 2013). Managing or treating the disorder requires better knowledge about the underlying mechanism. A large number of different possible molecular causes of CS theories have been proposed (reviewed in Brooks, 2013). Here, we have added significant evidence to support the idea that imperfect gene regulation, rather than DNA repair defects, underlies CS neurologic disease. The finding that CSB's function in neuronal differentiation and neuritogenesis can be, at least partially, bypassed in cell culture is encouraging, because it points to both a disease mechanism and possible pharmacological intervention. Crucially, we find that SYT9 and the secreted cargoes of the dense core vesicles it regulates (including BDNF) are, indeed, among the most downregulated in cerebella

OPEN ACCESS
Cell Press

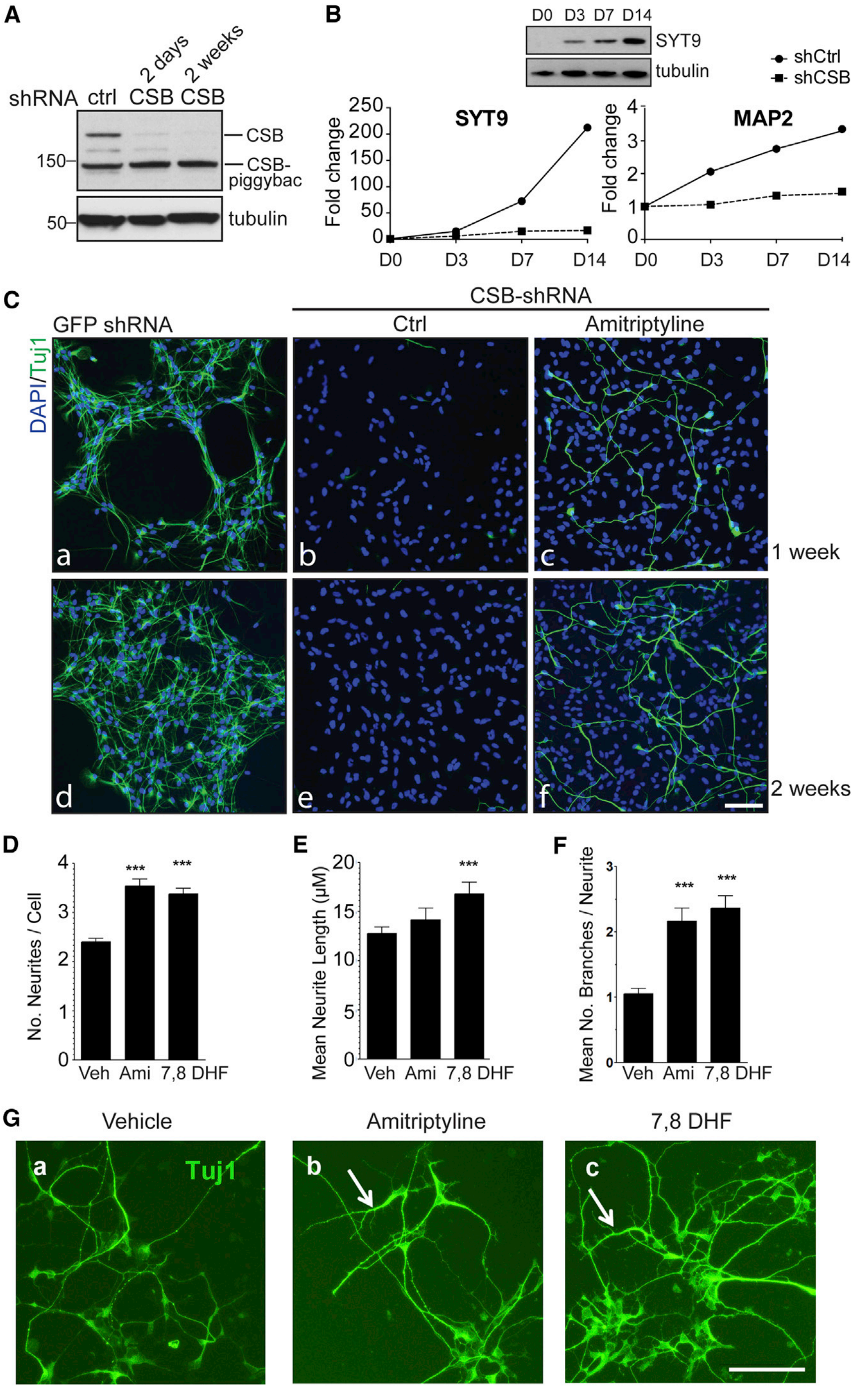

Figure 4. CSB Function Can Also Be Bypassed in Human Neural Stem Cells ReNcell VM and CSB iPSC Cells

(A) Western blot analysis of total extracts from ReNcell VM cells stably transduced with lentivirus carrying shCSB or control shRNA (shCtrl) for the indicated times. Note that the CSB-PiggyBac fusion protein (Bailey et al., 2012) is not affected by this CSB shRNA.

(B) Gene expression levels over time after serum withdrawal in WT (shCtrl) and CSB-depleted ReNcell VM cells (shCSB) assessed by qRT-PCR. Primers are given in Table S3. A western blot showing SYT9 levels is also shown.

(C) WT (GFP shRNA), and CSB-depleted ReNcell VM cells treated with amitriptyline (100 nM) or DMSO solvent alone (DMSO ctrl) were stained for Tuj1 (green) and DAPI (blue), 1 or 2 weeks after serum withdrawal. Scale bar, 100 μm.

(D) CSB iPSC-derived neurons were treated with amitriptyline (50 nM), 7,8-DHF (50 nM), or vehicle for 1 week and immunostained with Tuj1 antibodies. Quantitation of the number of neurites per cell. Bars represent mean ± SEM (n > 500 cells per data point). ***p < 0.0001.

(E) Same as in (D), but quantitation of neurite length per neuron. ***p < 0.0001.

(F) Same as in (D), but quantitation of neurite branches per neurite. ***p < 0.0001.

(G) Representative image of CSB iPSC-derived neurons treated with vehicle, amitriptyline or 7,8-DHF for 1 week. Arrows indicate regions with neurite branching in drug-treated cells, rarely observed in controls. Scale bar, 100 μm.

See also Table S3.

and cerebrum from CS patients. In general, the concurrent downregulation of so many genes involved in regulated exocytosis is likely to signify disruption of an entire gene regulatory network encompassing these genes.

Interestingly, work by Crabtree and co-workers has shown that another chromatin remodeler, BAF, also plays an important role in neuronal differentiation through an affect on gene regulatory networks, and microRNAs in particular (Sun et al., 2013). This led to the discovery that cellular reprogramming and neuronal differentiation can be induced by the ectopic expression of such microRNAs (Yoo et al., 2011). Numerous examples of induced cell differentiation in culture (cellular reprogramming) have now been described (reviewed in Amamoto and Arlotta, 2014). Induced differentiation in such systems is invariably brought about by artificially expressing transcription factor cocktails, and/or microRNAs; however, to the best of our knowledge, driving differentiation via proteins enabling exocytosis has not previously been reported. Given that BDNF and its pharmacological mimics, amitriptyline and 7,8-DHF, can also compensate for CSB deficiency, we speculate that increased expression of SYT9 leads to increased biogenesis, transport to release sites, and/or exocytosis of dense core vesicles, allowing augmented neurotrophin release and signaling. This would, in turn, help compensate for sub-optimal gene expression levels in CSB-deficient cells, helping to push through the gene expression changes required to allow differentiation. It is an exciting possibility that efficient cellular reprogramming might more

generally be achieved via stimulation of dense core vesicle release, which might drive differentiation in other cell and tissue systems as well.

While *Csb*$^{-/-}$ mice do not display the severe phenotypes that might have been expected from the human disease, we note that *Bdnf*$^{-/-}$ mice have decreased lifespan with nervous system dysfunction, including ataxia. Although their CNSs show no gross structural abnormalities, these mice also have substantially reduced numbers of cranial and spinal sensory neurons, and myelination defects (Ernfors et al., 1994; Jones et al., 1994; Cellerino et al., 1997), similar to what is observed in CS patients.

It is worth mentioning that, even though neuronal differentiation experiments with CSA-deficient cells could not be performed due to inefficient knockdown of CSA, we have previously reported that the gene expression defects observed in CSB fibroblasts are also observed in cells from CSA patients (Wang et al., 2014). Therefore, it seems reasonable to expect that CSA-deficient cells will show neuronal differentiation defects, and responses to drugs, that are similar to those of the CSB-deficient cells used here.

Arguably, the most exciting result of this study is the finding that the defects in neuronal differentiation and neuritogenesis observed in cells depleted for CSB can be partially bypassed by the addition of amitriptyline or 7,8-DHF. Amitriptyline is one of the most widely prescribed prophylactic medications for severe pediatric migraine (O'Brien et al., 2015). Unfortunately, its safety profile in CS patients has not been established, but given the limited treatment options available to these patients, we suggest that such trials be given high priority.

EXPERIMENTAL PROCEDURES

Cell Lines

SH-SY5Y cells were maintained in DMEM: Ham's F12 medium (1:1) supplemented with 15% fetal bovine serum (FBS) and 1% (v/v) penicillin/streptomycin and maintained in a 37°C incubator with 5% CO_2. ReNcell VM human neural stem cells were purchased from EMD Millipore. They were plated onto laminin (2 μg/ml, L2020, Sigma-Aldrich)-coated cell culture flasks and maintained in ReNcell NSC maintenance media (SCM005, EMD Millipore) supplemented with 20 μg/ml epidermal growth factor (EGF) and 20 μg/ml basic fibroblast growth factor (bFGF) (GF144, GF003, EMD Millipore). Cell culture media were changed every other day until cells were confluent.

Lentiviral Vectors

CSB-KD SH-SY5Y cells were described previously (Wang et al., 2014). The lentiviral plasmids (pLKO.1) encoding shRNAs against CSB (target sequence: 5′-TTATTCCATCTCCTTAACCGC3-′) or GFP (target sequence: 5′-GCAAGCT GACCCTGAAGTTCAT3-′) were from GE Healthcare, Dharmacon. Lentiviral particles were packaged in HEK293T cells. To transduce the ReNcell VM cells with the constructs, 50–100 μl viral solution (1 × 10^6 transducing units (TU)/ml) was added to 75%–85% confluent proliferating ReNcell VM cells in six-well dishes, incubated for 24 hr, and selected with puromycin (1 μg/ml) for 3–5 days.

For the rescue experiments, lentivirus constructs expressing human SYT9 cDNA, human DCN cDNA, human KPNA1 cDNA, and shRNAs against human IL2 (target sequence: 5′-TATTGCTGATTAAGTCCCTGGG-3′) were purchased from DharmaconGE. To transduce the CSB-KD SH-SY5Y cells, 50–100 μl viral solution (1 × 10^6 TU/ml) were added to 50% confluent cells in six-well dishes and incubated for 24 hr, and medium was replaced by differentiation medium.

For the ChIP assay, a lentiviral construct expressing human CSB cDNA was purchased from Applied Biological Materials (ABM) (http://www.abmgood.com). To transduce the WT SH-SY5Y cells, 500 μl viral solution (1 × 10^6 TU/ml) was added to 50% confluent cells in 10-cm dishes and incubated for 24 hr before changing the medium with differentiation medium.

In Vitro Differentiation

SH-SY5Y cells were plated onto poly-L-lysine (50 μg/ml)-coated plates and differentiated in the presence of reduced serum (0.5% FBS) and 10 μM RA as previously described (Wang et al., 2014).

ReNcell VM cells were plated onto laminin (2 μg/ml)-coated six-well plates and maintained in ReNcell NSC maintenance media supplemented with EGF (20 μg/ml) and bFGF (20 μg/ml) for 24 hr, and the cell culture medium was then replaced by differentiation medium and refreshed every 3 days for 1–4 weeks. In some experiments, BDNF (50 ng/ml or 3.6 nM) (Peprotech) was added with 10 μM RA to CSB-depleted cells.

For TrkB agonist and antagonist treatment in SH-SY5Y and ReNcell VM cells, 7,8-DHF hydrate, amitriptyline hydrochloride, and ANA-12 were purchased from Sigma-Aldrich and dissolved in DMSO (for 7,8-DHF hydrate) or H_2O (for amitriptyline and ANA-12) to generate 20 mM stock solutions; GA was dissolved in ethanol to make a 20 mM stock solution. Cells were treated with various concentrations of the agonists and antagonists as indicated.

Immunofluorescence

Cells were fixed and immunostained as previously described (Wang et al., 2014). The following primary and secondary antibodies were used: rabbit anti-Tuj1 monoclonal (1:1,000, MRB-435P, Covance) and Alexa Fluor 488 anti-rabbit (1:1,000, Life Technologies). Coverslips were counterstained with DAPI and mounted on slides using mounting medium with DAPI (Vector Laboratories). Images were acquired on a Zeiss Axio Imager M1 microscope equipped with an ORCA-ER camera (Hamamatsu) and controlled by Velocity 5.5.1 software. For cell counts, fluorescent images of the cells were collected using a 10× objective lens. At least 500 cells per condition and experiment were analyzed.

Western Blots

Whole-cell lysates were prepared as previously described (Wang et al., 2014). Primary antibodies were used at the following dilutions: anti-CSB (1:1,000, A301-345A, Bethyl Laboratories); anti-SYT9 (1:1,000, NBP1-92472, Novus Biologicals); anti-MAP2 (1:1,000, MAB3418, EMD); anti-Tyrosine Hydroxylase (1:500, AB152, EMD); anti-α-tubulin (1:10,000, ab15246, Abcam); anti-TrkB (1:1,000, 4606S, Cell Signaling); and anti-phospho-TrkB Tyr706 (1:500, C50F3, Cell Signaling).

ChIP-qPCR

ChIP was performed as described elsewhere (Saponaro et al., 2014). Briefly, cells were cross-linked with 1% formaldehyde solution for 15 min at room temperature. Cross-linking was quenched by the addition of glycine to a final concentration of 125 mM. Samples were sonicated to generate DNA fragments of less than 500 bp. For immunoprecipitation, 1 μg of GFP antibody (ab290, Abcam) or 2 μg of RNAP II antibody (N-20, Santa Cruz Biotechnology) was added to the cleared chromatin and incubated overnight at 4°C in the presence of 25 μl slurry of protein G-sepharose beads. Immunoprecipitated DNA was quantified by real-time PCR. Primers sequences used in qPCR are described in Table S3.

RNA Extraction, qRT-PCR, and DNA Microarray

Total RNA was prepared as described earlier (Wang et al., 2014). For qRT-PCR, complementary DNAs from 100 ng of total RNAs were synthesized using the TaqMan Reverse Transcription Reagents Kit (Life Technologies). Real-time qPCR was carried out with the iQ-SYBR Green Master Mix (BioRad) using the CFX96 Real-Time PCR system (BioRad). Gene expression levels were normalized against GAPDH and 18S rRNA levels in each sample, and the fold changes were calculated by setting the expression levels of each gene in control cells as 1. Primer sequences are listed in Table S3. We performed RNA isolation from tissues and DNA microarray as previously described (Wang et al., 2014).

For microarray experiments, double-stranded cDNAs were synthesized from 10 μg of total RNA using the cDNA synthesis kit according to the Roche

user protocol. Up to 1 μg of double-stranded cDNA was labeled with Cy3 and hybridized on a NimbleGen 12 × 125K Human Expression array, followed by washing and drying according to the manufacturer's instructions (NimbleGen, Roche). Arrays were scanned with a NimbleGen MS200 micro-array scanning system, and data acquisition was performed with NimbleScan according to the manufacturer's protocols.

Neuronal Differentiation of CSB iPSCs

iPSCs from CSB patient GM00739 (Andrade et al., 2012) were cultured in mTeSR1 media (Stem Cell Technologies) on Matrigel (Corning)-coated cultureware, with daily media changes. Neural differentiation was performed according to published methods (Brennand et al., 2011), with minor modifications. Cells from iPSC colonies were harvested using Accutase (Sigma), and embryoid bodies were formed by seeding single cells into Aggrewell 800 plates for 24 hr in DMEM/F12 media supplemented with N2 supplement, non-essential amino acids, 2 μg/ml heparin and penicillin/streptomycin (all from Life Technologies), as well as 10 ng/ml fibroblast growth factor 2 and 2 μM dosomorphin (both from Sigma), 10 μM SB431542 (Stemgent), and 10 μM Y-27632 (Stem Cell Technologies). Cells were allowed to form embryoid bodies for 24 hr inside the Aggrewells, and then they were transferred to fresh media without 10 μM Y-27632 in low-attachment six-well plates (Corning). Embryoid bodies were grown for 4 days, with daily media changes. Embryoid bodies were then transferred to six-well plates coated with 0.1 mg/ml poly-L-ornithine (Sigma) and 20 μg/ml laminin (Life Technologies) to form rosettes. Rosette media consisted of DMEM/F12 media supplemented with N2 supplement, non-essential amino acids, 2 μg/ml heparin, B27 supplement without vitamin A, penicillin/streptomycin, 1 μg/ml laminin (all from Life Technologies), and 10 ng/ml fibroblast growth factor 2 (Sigma). Rosettes were grown for 1 week, with media changes every other day until the appearance of rosette structures. Rosettes were harvested manually and dissociated with Accutase (Sigma). The resultant neural precursors were grown for two passages on six-well plates in neural precursor media; this was identical to rosette media, except that the concentration of fibroblast growth factor 2 was increased to 20 ng/ml (Sigma). Neural precursors were grown on six-well plates coated with 0.1 mg/ml poly-L-ornithine (Sigma) and 10 μg/ml laminin (Life Technologies), with media changes every other day and passages every 5 days. In order to induce neuronal differentiation, cells were harvested with Accutase and grown in neuronal differentiation media. Differentiation media consisted of neurobasal media supplemented with N2 supplement and B27 supplement lacking vitamin A, glutaMAX, penicillin/streptomycin and 1 μg/ml laminin (all from Life Technologies), 1 μM dibutyryl cyclic AMP and 200 ng/ml ascorbic acid (both from Sigma), 10 ng/ml BDNF, 10 ng/ml glial-cell-derived neurotrophic factor (GDNF) and 10 ng/ml insulin-like growth factor 1 (IGF-1) (all from Peprotech). Cells were cultured in ibidi μ-Slide eight-well slides (ibidi USA). At the time of seeding, cells were cultured in the presence of 50 nM amitriptyline, 50 nM 7,8-DHF or saline vehicle. Drug treatments were performed in duplicate wells and on duplicate slides. Media and drugs were changed every other day, and after 1 week of drug treatment, cultures were fixed with 4% paraformaldehyde. As a separate experiment, an additional vial of CSB iPSCs was processed through the full differentiation protocol several weeks later to confirm reproducibility. Fixed cells were stained overnight with antibodies against Tuj1 (1:1,000, Stem Cell Technologies) followed by goat anti-mouse antibodies conjugated to Alexa Fluor 488 (1:1,000, Life Technologies) and mounting media containing DAPI (Vector Laboratories). Images were obtained with an inverted fluorescence microscope (AxioVert, Zeiss) using Velocity software (PerkinElmer), with the imaging analyst blinded to treatment group. Neuronal morphology was quantified using MetaMorph software equipped with the neurite outgrowth module (Molecular Devices), with the imaging analyst blinded to treatment group. 500–800 cells were analyzed per treatment group. For statistical testing, neurite number and branching were analyzed with a one-way ANOVA, followed by Bonferroni post hoc testing. Neurite length was analyzed with the Kruskal-Wallis test, followed by Dunn's post hoc test. Statistical testing was performed with GraphPad Prism 5 software (GraphPad Software). Differences were considered statistically significant at $p < 0.05$.

Transcriptome Analysis

Cerebrum samples from CS patients and age-matched controls were provided by the NIH NeuroBioBank (NBB). The clinical history of patients was described previously (Wang et al., 2014)). Total RNA from ~100 mg of frozen brain tissue was isolated using the QIAGEN RNeasy Lipid Tissue Mini Kit. RNA concentration and integrity (RIN) were assessed by Agilent Bioanalyzer Nano ChIP. Double-stranded cDNA synthesis and DNA microarray was performed as described earlier (see also Wang et al., 2014).

DNA Microarray Statistic Analysis

Data were analyzed using Bioconductor 2.12 (http://www.bioconductor.org) running on R 3.0.0. Raw pair files (NimbleGen *Homo sapiens* HG18 expression array) were quantile normalized in R. Differential gene expression was assessed using an empirical Bayes t test (Limma package). The p values were adjusted for multiple testing using the Benjamini-Hochberg method (Smyth, 2005). Any probe sets that exhibited an adjusted p value of 0.05 were defined as differentially expressed. In addition, any probe sets that exhibited an absolute fold change of >1.5 were used to generate a heatmap.

ACCESSION NUMBERS

The accession numbers for the lentivirus constructs reported in this paper are as follows: European Nucleotide Archive: BC046367 (for constructs expressing human SYT9 cDNA), European Nucleotide Archive: BC005322 (for constructs expressing human DCN cDNA), and European Nucleotide Archive: BC002374 (for constructs expressing human KPNA1 cDNA). The accession number for the data reported in this paper is GEO: GSE58072.

SUPPLEMENTAL INFORMATION

Supplemental Information includes eight figures and three tables and can be found with this article online at http://dx.doi.org/10.1016/j.celrep.2016.02.051.

AUTHOR CONTRIBUTIONS

Y.W. performed all experiments, except those in Figures 4D–4G, which were performed by J.J.-T. and R.R.L., using iPSCs developed by A.M. P.C. and A.S. performed bioinformatics analysis. R.L. and J.Q.S. supervised the work. J.Q.S. wrote the paper, with input from all authors.

ACKNOWLEDGMENTS

This work was supported by a grant from the European Research Council and by the Francis Crick Institute (grant number FCI01), which receives its core funding from Cancer Research UK, the UK Medical Research Council, and the Wellcome Trust. The NICHD Brain and Tissue Bank for Developmental Disorders of the University of Maryland, Baltimore (member of NIH NBB) kindly provided brain tissue samples. The Crick Institute's Advanced Sequencing Facility and the Cell Services Facility are thanked for their expert assistance. Giampietro Schiavo, P.J. Brooks, Peter Verrijzer, Laura Williamson, Michael Lim, and Barbara Dirac-Svejstrup are thanked for comments.

Received: July 9, 2015
Revised: December 22, 2015
Accepted: February 8, 2016
Published: March 10, 2016

REFERENCES

Amamoto, R., and Arlotta, P. (2014). Development-inspired reprogramming of the mammalian central nervous system. Science *343*, 1239882.

Andrade, L.N., Nathanson, J.L., Yeo, G.W., Menck, C.F., and Muotri, A.R. (2012). Evidence for premature aging due to oxidative stress in iPSCs from Cockayne syndrome. Hum. Mol. Genet. *21*, 3825–3834.

Bailey, A.D., Gray, L.T., Pavelitz, T., Newman, J.C., Horibata, K., Tanaka, K., and Weiner, A.M. (2012). The conserved Cockayne syndrome B-piggyBac fusion protein (CSB-PGBD3) affects DNA repair and induces both interferon-like and innate antiviral responses in CSB-null cells. DNA Repair (Amst.) *11*, 488–501.

OPEN ACCESS
CellPress

Brennand, K.J., Simone, A., Jou, J., Gelboin-Burkhart, C., Tran, N., Sangar, S., Li, Y., Mu, Y., Chen, G., Yu, D., et al. (2011). Modelling schizophrenia using human induced pluripotent stem cells. Nature *473*, 221–225.

Brooks, P.J. (2013). Blinded by the UV light: how the focus on transcription-coupled NER has distracted from understanding the mechanisms of Cockayne syndrome neurologic disease. DNA Repair (Amst.) *12*, 656–671.

Cellerino, A., Carroll, P., Thoenen, H., and Barde, Y.A. (1997). Reduced size of retinal ganglion cell axons and hypomyelination in mice lacking brain-derived neurotrophic factor. Mol. Cell. Neurosci. *9*, 397–408.

Ciaffardini, F., Nicolai, S., Caputo, M., Canu, G., Paccosi, E., Costantino, M., Frontini, M., Balajee, A.S., and Proietti-De-Santis, L. (2014). The cockayne syndrome B protein is essential for neuronal differentiation and neuritogenesis. Cell Death Dis. *5*, e1268.

Citterio, E., Rademakers, S., van der Horst, G.T., van Gool, A.J., Hoeijmakers, J.H., and Vermeulen, W. (1998). Biochemical and biological characterization of wild-type and ATPase-deficient Cockayne syndrome B repair protein. J. Biol. Chem. *273*, 11844–11851.

Cong, W.N., Chadwick, W., Wang, R., Daimon, C.M., Cai, H., Amma, J., Wood, W.H., 3rd, Becker, K.G., Martin, B., and Maudsley, S. (2015). Amitriptyline improves motor function via enhanced neurotrophin signaling and mitochondrial functions in the murine N171-82Q Huntington disease model. J. Biol. Chem. *290*, 2728–2743.

Dean, C., Dunning, F.M., Liu, H., Bomba-Warczak, E., Martens, H., Bharat, V., Ahmed, S., and Chapman, E.R. (2012). Axonal and dendritic synaptotagmin isoforms revealed by a pHluorin-syt functional screen. Mol. Biol. Cell *23*, 1715–1727.

Dimos, J.T., Rodolfa, K.T., Niakan, K.K., Weisenthal, L.M., Mitsumoto, H., Chung, W., Croft, G.F., Saphier, G., Leibel, R., Goland, R., et al. (2008). Induced pluripotent stem cells generated from patients with ALS can be differentiated into motor neurons. Science *321*, 1218–1221.

Encinas, M., Iglesias, M., Liu, Y., Wang, H., Muhaisen, A., Ceña, V., Gallego, C., and Comella, J.X. (2000). Sequential treatment of SH-SY5Y cells with retinoic acid and brain-derived neurotrophic factor gives rise to fully differentiated, neurotrophic factor-dependent, human neuron-like cells. J. Neurochem. *75*, 991–1003.

Ernfors, P., Lee, K.F., and Jaenisch, R. (1994). Mice lacking brain-derived neurotrophic factor develop with sensory deficits. Nature *368*, 147–150.

Iyama, T., Lee, S.Y., Berquist, B.R., Gileadi, O., Bohr, V.A., Seidman, M.M., McHugh, P.J., and Wilson, D.M., 3rd. (2015). CSB interacts with SNM1A and promotes DNA interstrand crosslink processing. Nucleic Acids Res. *43*, 247–258.

Jang, S.W., Liu, X., Yepes, M., Shepherd, K.R., Miller, G.W., Liu, Y., Wilson, W.D., Xiao, G., Blanchi, B., Sun, Y.E., and Ye, K. (2010). A selective TrkB agonist with potent neurotrophic activities by 7,8-dihydroxyflavone. Proc. Natl. Acad. Sci. USA *107*, 2687–2692.

Jiang, M., Peng, Q., Liu, X., Jin, J., Hou, Z., Zhang, J., Mori, S., Ross, C.A., Ye, K., and Duan, W. (2013). Small-molecule TrkB receptor agonists improve motor function and extend survival in a mouse model of Huntington's disease. Hum. Mol. Genet. *22*, 2462–2470.

Jones, K.R., Fariñas, I., Backus, C., and Reichardt, L.F. (1994). Targeted disruption of the BDNF gene perturbs brain and sensory neuron development but not motor neuron development. Cell *76*, 989–999.

O'Brien, H.L., Kabbouche, M.A., Kacperski, J., and Hershey, A.D. (2015). Treatment of pediatric migraine. Curr. Treat. Options Neurol. *17*, 326.

Park, H., and Poo, M.M. (2013). Neurotrophin regulation of neural circuit development and function. Nat. Rev. Neurosci. *14*, 7–23.

Saponaro, M., Kantidakis, T., Mitter, R., Kelly, G.P., Heron, M., Williams, H., Söding, J., Stewart, A., and Svejstrup, J.Q. (2014). RECQL5 controls transcript elongation and suppresses genome instability associated with transcription stress. Cell *157*, 1037–1049.

Scheibye-Knudsen, M., Croteau, D.L., and Bohr, V.A. (2013). Mitochondrial deficiency in Cockayne syndrome. Mech. Ageing Dev. *134*, 275–283.

Smyth, G. (2005). Limma: linear models for microarray data. In Bioinformatics and Computational Biology Solutions using R and Bioconductor, R. Gentleman, V. Carey, S. Dudoit, R. Irizarry, and W. Huber, eds. (New York: Springer), pp. 397–420.

Spivak, G. (2005). UV-sensitive syndrome. Mutat. Res. *577*, 162–169.

Sun, A.X., Crabtree, G.R., and Yoo, A.S. (2013). MicroRNAs: regulators of neuronal fate. Curr. Opin. Cell Biol. *25*, 215–221.

Troelstra, C., van Gool, A., de Wit, J., Vermeulen, W., Bootsma, D., and Hoeijmakers, J.H. (1992). ERCC6, a member of a subfamily of putative helicases, is involved in Cockayne's syndrome and preferential repair of active genes. Cell *71*, 939–953.

van der Horst, G.T., van Steeg, H., Berg, R.J., van Gool, A.J., de Wit, J., Weeda, G., Morreau, H., Beems, R.B., van Kreijl, C.F., de Gruijl, F.R., et al. (1997). Defective transcription-coupled repair in Cockayne syndrome B mice is associated with skin cancer predisposition. Cell *89*, 425–435.

Vélez-Cruz, R., and Egly, J.M. (2013). Cockayne syndrome group B (CSB) protein: at the crossroads of transcriptional networks. Mech. Ageing Dev. *134*, 234–242.

Vermeulen, W., and Fousteri, M. (2013). Mammalian transcription-coupled excision repair. Cold Spring Harb. Perspect. Biol. *5*, a012625.

Vessoni, A.T., Herai, R.H., Karpiak, J.V., Leal, A.M., Trujillo, C.A., Quinet, A., Agnez Lima, L.F., Menck, C.F., and Muotri, A.R. (2016). Cockayne Syndrome-derived neurons display reduced synapse density and altered neural network synchrony. Hum Mol Genet. Published online January 10, 2016. http://dx.doi.org/10.1093/hmg/ddw008.

Wang, Y., Chakravarty, P., Ranes, M., Kelly, G., Brooks, P.J., Neilan, E., Stewart, A., Schiavo, G., and Svejstrup, J.Q. (2014). Dysregulation of gene expression as a cause of Cockayne syndrome neurological disease. Proc. Natl. Acad. Sci. USA *111*, 14454–14459.

Weidenheim, K.M., Dickson, D.W., and Rapin, I. (2009). Neuropathology of Cockayne syndrome: Evidence for impaired development, premature aging, and neurodegeneration. Mech. Ageing Dev. *130*, 619–636.

Xu, J., Mashimo, T., and Südhof, T.C. (2007). Synaptotagmin-1, -2, and -9: Ca(2+) sensors for fast release that specify distinct presynaptic properties in subsets of neurons. Neuron *54*, 567–581.

Yoo, A.S., Sun, A.X., Li, L., Shcheglovitov, A., Portmann, T., Li, Y., Lee-Messer, C., Dolmetsch, R.E., Tsien, R.W., and Crabtree, G.R. (2011). MicroRNA-mediated conversion of human fibroblasts to neurons. Nature *476*, 228–231.

Zhao, S., Gao, X., Dong, W., and Chen, J. (2015). The role of 7,8-dihydroxyflavone in preventing dendrite degeneration in cortex after moderate traumatic brain injury. Mol. Neurobiol. Published online March 24, 2015. http://dx.doi.org/10.1007/s12035-015-9128-z.

Zhu, D., Zhou, W., Liang, T., Yang, F., Zhang, R.Y., Wu, Z.X., and Xu, T. (2007). Synaptotagmin I and IX function redundantly in controlling fusion pore of large dense core vesicles. Biochem. Biophys. Res. Commun. *361*, 922–927.

Cell Reports, Volume *14*

Supplemental Information

Pharmacological Bypass of Cockayne Syndrome B Function in Neuronal Differentiation

Yuming Wang, Jace Jones-Tabah, Probir Chakravarty, Aengus Stewart, Alysson Muotri, Rebecca R. Laposa, and Jesper Q. Svejstrup

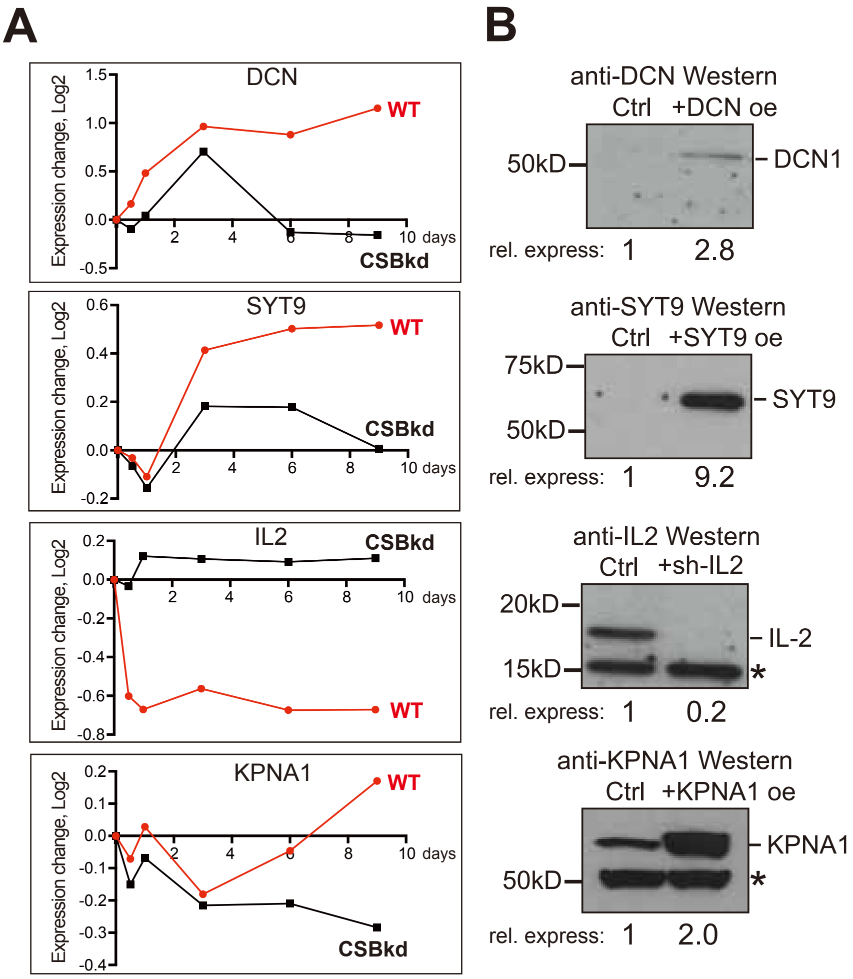
A
B
DCN
WT
CSBkd
SYT9
IL2
KPNA1
Expression change, Log2
days
anti-DCN Western
Ctrl
+DCN oe
50kD
DCN1
rel. express: 1 2.8
anti-SYT9 Western
+SYT9 oe
75kD
SYT9
rel. express: 1 9.2
anti-IL2 Western
+sh-IL2
20kD
15kD
IL-2
rel. express: 1 0.2
anti-KPNA1 Western
+KPNA1 oe
KPNA1
rel. express: 1 2.0

Figure S1

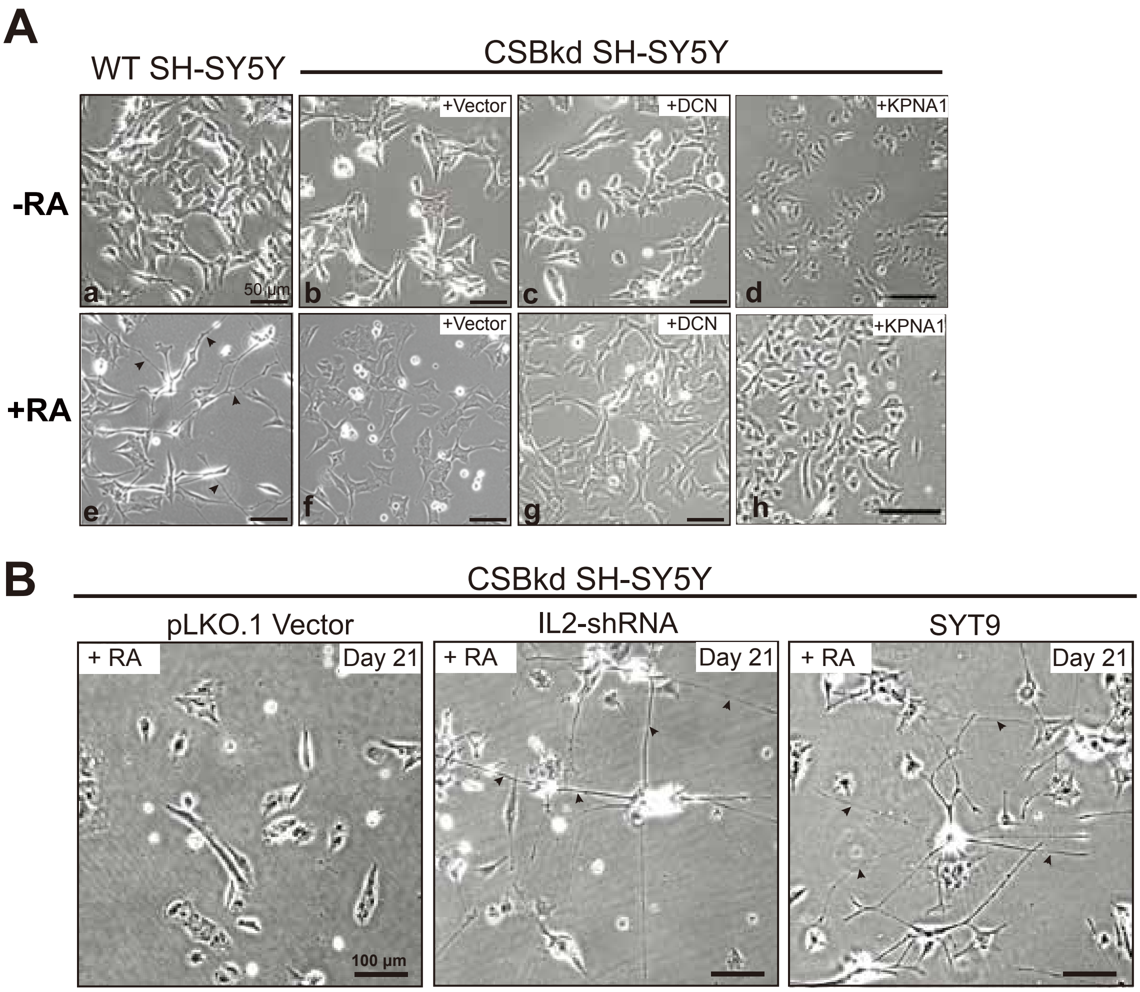
A
WT SH-SY5Y
CSBkd SH-SY5Y
-RA
+RA
+Vector
+DCN
+KPNA1
50 μm
a
b
c
d
e
f
g
h
B
CSBkd SH-SY5Y
pLKO.1 Vector
IL2-shRNA
SYT9
+ RA
Day 21
100 μm

Figure S2

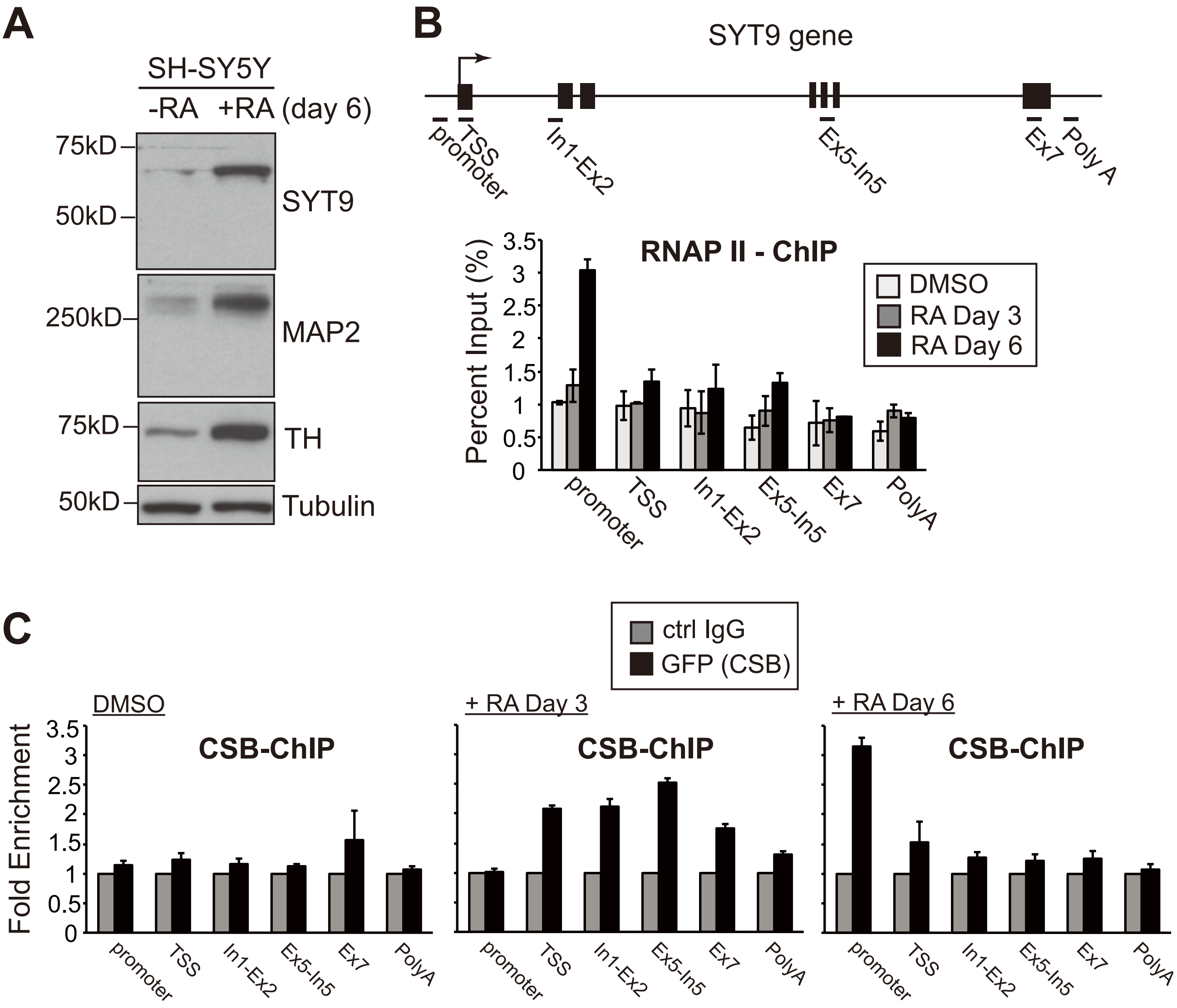
A
SH-SY5Y
-RA +RA (day 6)
75kD
50kD
SYT9
250kD
MAP2
75kD
TH
50kD
Tubulin
B
SYT9 gene
promoter
TSS
In1-Ex2
Ex5-In5
Ex7
PolyA
RNAP II - ChIP
DMSO
RA Day 3
RA Day 6
Percent Input (%)
C
ctrl IgG
GFP (CSB)
DMSO
+ RA Day 3
+ RA Day 6
CSB-ChIP
Fold Enrichment

Figure S3

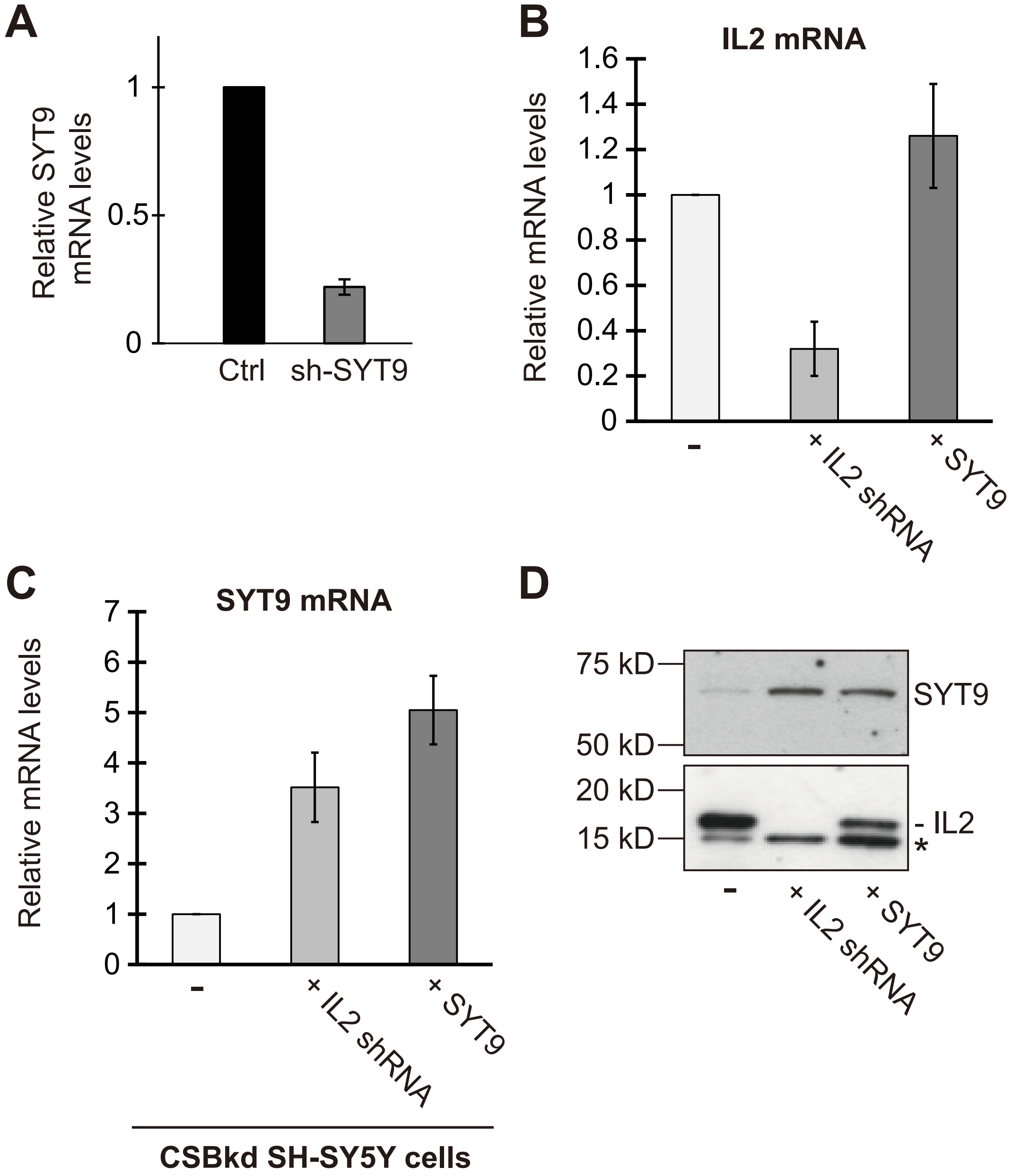
A
Relative SYT9 mRNA levels
1
0.5
0
Ctrl
sh-SYT9
B
IL2 mRNA
Relative mRNA levels
1.6
1.4
1.2
1
0.8
0.6
0.4
0.2
0
-
+ IL2 shRNA
+ SYT9
C
SYT9 mRNA
Relative mRNA levels
7
6
5
4
3
2
1
0
-
+ IL2 shRNA
+ SYT9
CSBkd SH-SY5Y cells
D
75 kD
50 kD
SYT9
20 kD
15 kD
- IL2
*
-
+ IL2 shRNA
+ SYT9

Figure S4

A

Gene symbol	Ensembl ID	Day 3 Fold-change*	Day 3 FDR-adjusted *P* value	Day 6 Fold-change*	Day 6 FDR-adjusted *P* value
SYT9	ENSG00000170743	6.16	4.32e-06	6.22	5.45e-07
ASPM	ENSG00000066279	2.61	8.30e-05	3.22	3.61e-06
EGFR	ENSG00000146648	2.14	0.000190	2.07	0.000115
SYT4	ENSG00000132872	2.28	0.001522	2.68	0.000174
GRIK4	ENSG00000149403	-	-	3.13	0.000195
PHOX2B	ENSG00000109132	-	-	2.07	0.000279
NEUROG2	ENSG00000178403	3.67	0.007165	6.17	0.000343
VPS16	ENSG00000215305	1.65	0.003025	1.82	0.000420
DBH	ENSG00000123454	2.40	0.000445	1.65	0.019542
NEUROG3	ENSG00000122859	2.37	0.010554	3.35	0.000604
LZTS1	ENSG00000061337	-	-	2.52	0.000708
ISL1	ENSG00000016082	-	-	2.08	0.000855
PTPN11	ENSG00000179295	2.12	0.001005	-	-
RND1	ENSG00000172602	-	-	2.13	0.001083
PEBP1	ENSG00000089220	1.74	0.012385	2.09	0.001213
TRKB	ENSG00000148053	-	-	2.38	0.001462
MYH10	ENSG00000133026	1.53	0.010630	1.71	0.001462
TUBB4	ENSG00000104833	2.02	0.002253	1.60	0.023046
CALCA	ENSG00000110680	2.25	0.004745	2.28	0.002937
CNP	ENSG00000173786	1.64	0.003020	-	-
ASCL1	ENSG00000139352	-	-	1.86	0.003074
SEMA4D	ENSG00000187764	1.71	0.020099	1.60	0.005496
ATF5	ENSG00000169136	-	-	2.20	0.006120
PHGDH	ENSG00000092621	-	-	2.00	0.006922
BDNF	ENSG00000176697	-	-	2.20	0.007249
TIMP2	ENSG00000035862	1.54	0.022951	1.59	0.007260
NEFH	ENSG00000100285	-	-	2.13	0.007971
EPHB3	ENSG00000182580	-	-	2.27	0.007991
CCND2	ENSG00000118971	2.28	0.011732	1.98	0.029496
NGRN	ENSG00000182768	-	-	1.87	0.012257
NRP2	ENSG00000118257	3.04	0.012886	-	-
NFASC	ENSG00000163531	-	-	1.72	0.013841
CDK5RAP2	ENSG00000136861	-	-	1.68	0.014350
FARP2	ENSG00000006607	-	-	1.80	0.016380
SERPINF1	ENSG00000132386	1.81	0.023189	2.00	0.021479
TUBB2B	ENSG00000137285	1.54	0.021551	-	-
KLF7	ENSG00000118263	1.61	0.044933	1.70	0.023644
PSEN1	ENSG00000080815	1.91	0.043057	1.97	0.031566
SEMA6A	ENSG00000092421	-	-	1.60	0.034519
EPHB2	ENSG00000133216	-	-	1.64	0.039308
MIB1	ENSG00000101752	1.60	0.029260	1.54	0.039811
LRRTM1	ENSG00000162951	-	-	1.56	0.049225
TGIF2	ENSG00000118707	-	-	1.54	0.049834

B

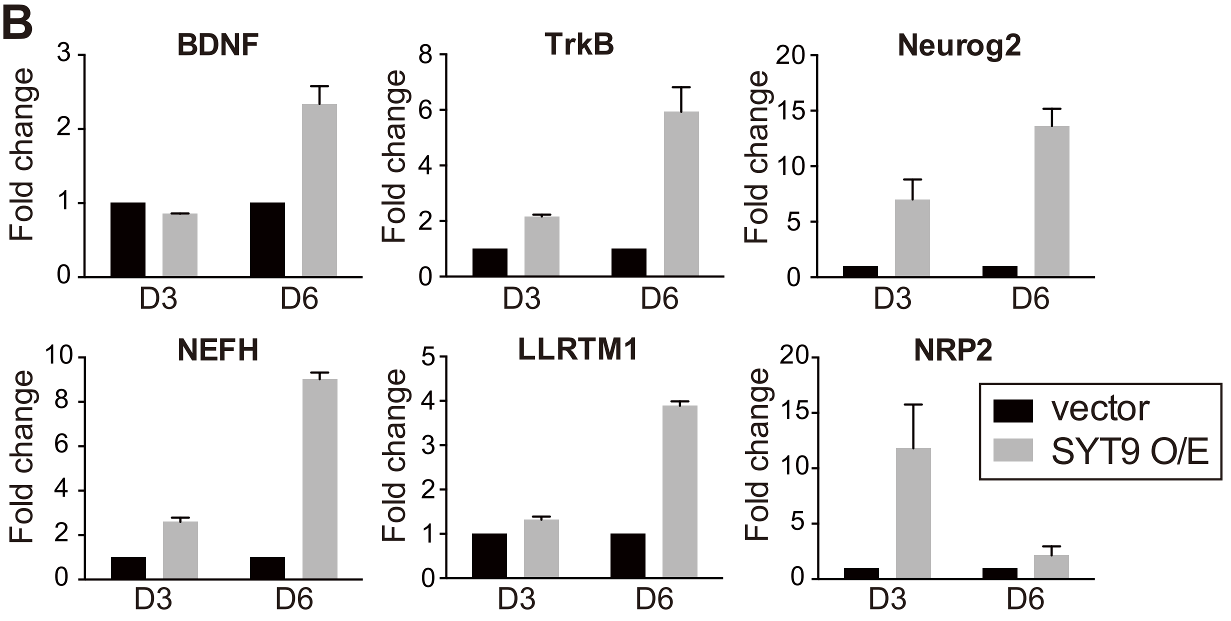

Figure S5

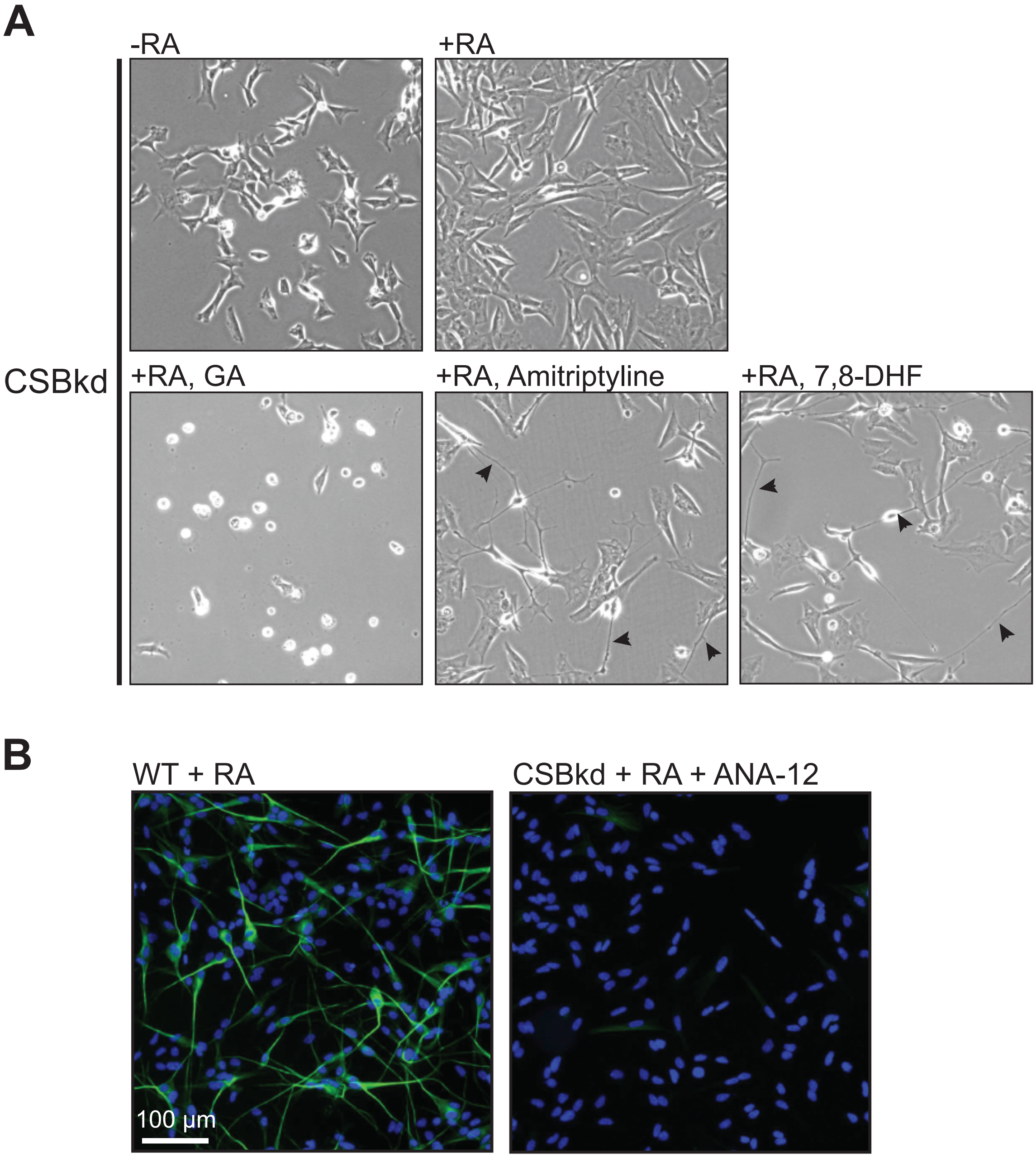
A
-RA
+RA
CSBkd
+RA, GA
+RA, Amitriptyline
+RA, 7,8-DHF
B
WT + RA
CSBkd + RA + ANA-12
100 µm

Figure S6

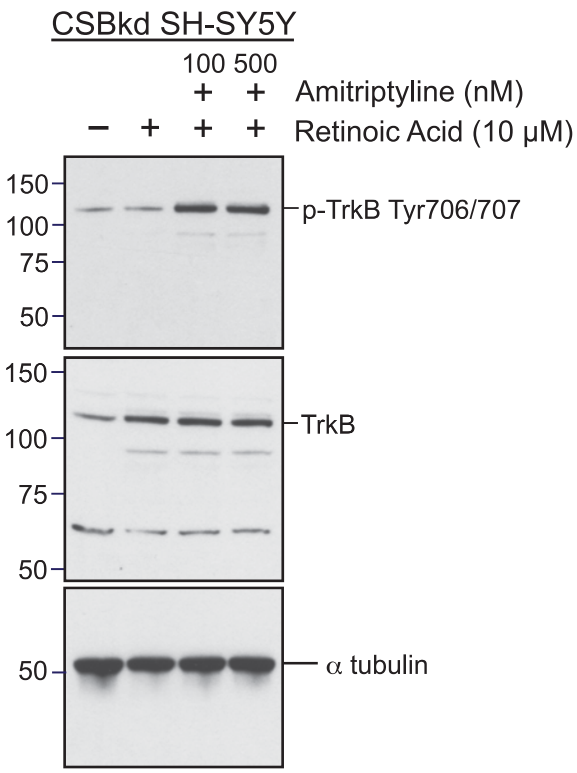
CSBkd SH-SY5Y
100 500
+ + Amitriptyline (nM)
− + + + Retinoic Acid (10 μM)
150
100
75
50
p-TrkB Tyr706/707
150
100
75
50
TrkB
50
α tubulin

Figure S7

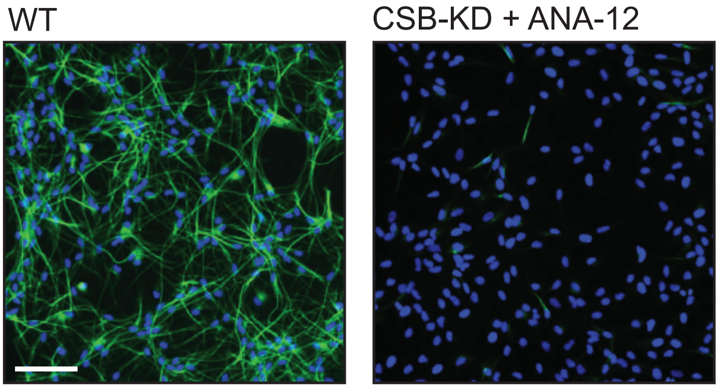

Figure S8

SUPPLEMENTARY FIGURE LEGENDS

Figure S1, related to Figure 1. qRT-PCR validation of selected genes during differentiation of WT and CSB-depleted SH-SY5Y cells. A. Quantitative RT-PCR of selected CSB-dependent genes identified by microarray expression analysis (Wang et al., 2014), at different times (in days) after retinoic acid addition. **B.** Western blot analysis of total extracts from CSB-depleted SH-SY5Y cells transduced with lentivirus carrying DCN-cDNA, SYT9-cDNA, IL2-shRNA, or KPNA1-DNA, and treated with retinoic acid for 6 days. Asterisks indicate non-specific bands.

Figure S2, related to Figure 1. Ectopic SYT9 expression or IL2 knockdown can restore neuritogenesis in CSB-depleted SH-SY5Y cells. A. CSB-depleted SH-SY5Y cells transduced with lentiviral particles containing empty vector, DCN-cDNA, or KPNA1-cDNA were treated with 10 μM RA, and phase contract images were taken on day 6 of differentiation. Left panel represents WT control cells. Arrowheads denote examples of neurite outgrowth. Scale bar = 50 μm. **B.** CSB-depleted SH-SY5Y cells expressing IL2-shRNA or SYT9-cDNA were cultured in differentiation media containing 10 μM RA. Phase contract images were taken on day 21. Arrowheads denote examples of neurite outgrowth. Scale bar = 100 μm.

Figure S3, related to Figure 1. Evidence that SYT9 induction is regulated by CSB at the level of transcription. A. Western blot analysis of whole cell lysates from undifferentiated (-RA) and differentiated (+RA) WT SH-SY5Y cells with antibodies against SYT9, MAP2, TH, and alpha tubulin. **B.** Upper, diagram showing the location of SYT9 primer sets used for ChIP-qPCR in cells expressing CSB-GFP.

Lower, ChIP analysis with antibody to RNAPII. **C.** CSB-GFP ChIP before and during RA treatment. Primers in Table S3.

Figure S4, related to Figure 1. Knockdown efficiency and effects thereof in SH-SY5Y cells. A. Quantification of SYT9 mRNA knockdown efficiency in SH-SY5Y cells stably transduced with lentivirus containing SYT9 shRNA. GFP-shRNA was used as control. **B.** Quantification of IL2 mRNA expression in CSB-depleted SH-SY5Y cells expressing IL2-shRNA, or SYT9 cDNA. Total RNA was extracted on day 3 after lentivirus transduction. **C.** As in (**B.**) but quantification of SYT9 mRNA. **D.** IL2 and SYT9 proteins levels, detected by Western blotting. Asterisk in IL2 blot indicates non-specific band.

Figure S5, related to Figure 2. SYT9-regulated genes identified by microarray analysis. A. List of genes (GO term 'Neurogenesis') that are up-regulated in CSB-depleted SH-SY5Y cells ectopically expressing SYT9 at day 3 and 6 after RA-addition. Fold-changes represent the average of two independent biological replicates. TRKB and BDNF are highlighted. **B.** RT-PCR validations in triplicate ± s.d. of each representative RNA sample. Primers used for RT-PCR in Table S3.

Figure S6, related to Figure 3. TRKB antagonists fail to rescue the differentiation defects of CSB-depleted SH-SY5Y cells. A. CSB-depleted SH-SY5Y cells were treated with either gambogic acid (GA, 100 nM), amitriptyline (100 nM) or 7,8-DHF (100 nM), together with 10 μM RA, and phase contract images were taken on day 6 of differentiation. As suggested by the pictures, GA-treated cultures suffered significant cell death. **B.** CSB-depleted SH-SY5Y cells were treated with RA

(10 μM) and ANA-12 (100 nM), and stained for Tuj1 (green) and DAPI (blue) on day 6 after treatment. WT cells (GFP-shRNA) are shown in the left panel for comparison. Scale bar = 100 μm.

Figure S7, related to Figure 3. Effects of amitriptyline on the expression of TRKB and phospho-TrkB. Western blot analysis of total extracts from CSB-depleted SH-SY5Y cells treated with RA (10 μM) and amitriptyline (100 nM or 500 nM) for 6 days before lysis. Upper panel, pTrkB-specific antibody.

Figure S8, related to Figure 4. ANA-12 failed to rescue the defects of CSB-KD ReNcell VM cell differentiation. WT and CSB-depleted ReNcell VM cells treated with ANA-12 (100 nM) were stained for Tuj1 (green) and DAPI (blue) on week 2 after serum withdrawal. WT cells treated with RA are shown for comparison. Scale bar = 100 μm.

Supplementary Tables

• 3 Supplementary Tables (Table S1-S3)
Table S1 is related to Figure 2: **Up- and down-regulated genes in CSBkd SH-SY5Y cells ectopically expressing SYT9**

Table S2 is related to Figure 3: **Genes that are significanctly up- or down-regulated in CS patients' cerebrum**

Table S3 is related to Experimental procedures: **Primers used for qRT-PCR validation of microarray data**
